# Identification and Validation Model for Informative Liquid Biopsy-Based microRNA Biomarkers: Insights from Germ Cell Tumor In Vitro, In Vivo and Patient-Derived Data

**DOI:** 10.3390/cells8121637

**Published:** 2019-12-14

**Authors:** João Lobo, Ad J.M. Gillis, Annette van den Berg, Lambert C. J. Dorssers, Gafanzer Belge, Klaus-Peter Dieckmann, Henk P. Roest, Luc J. W. van der Laan, Jourik Gietema, Robert J. Hamilton, Carmen Jerónimo, Rui Henrique, Daniela Salvatori, Leendert H. J. Looijenga

**Affiliations:** 1 Princess Máxima Center for Pediatric Oncology, Heidelberglaan 25, 3584 CS Utrecht, The Netherlands; 2 Department of Pathology, Portuguese Oncology Institute of Porto (IPOP), R. Dr. António Bernardino de Almeida, 4200-072 Porto, Portugal; henrique@ipoporto.min-saude.pt; 3 Cancer Biology and Epigenetics Group, Research Center of Portuguese Oncology Institute of Porto (GEBC CI-IPOP) and Porto Comprehensive Cancer Center (P.CCC), R. Dr. António Bernardino de Almeida, 4200-072 Porto, Portugal; carmenjeronimo@ipoporto.min-saude.pt; 4 Department of Pathology and Molecular Immunology, Institute of Biomedical Sciences Abel Salazar, University of Porto (ICBAS-UP), Rua Jorge Viterbo Ferreira 228, 4050-513 Porto, Portugal; 5 Department of Pathology, Lab. for Exp. Patho-Oncology (LEPO), Erasmus MC-University Medical Center Rotterdam, Cancer Institute, Be-432A, PO Box 2040, 3000 CA Rotterdam, The Netherlands; l.dorssers@erasmusmc.nl; 6 University Bremen, Faculty of Biology & Chemistry, Bibliothekstraße 1, 28359 Bremen, Germany; belge@uni-bremen.de; 7 Department of Urology, Hodentumorzentrum Hamburg, Asklepios Klinik Altona, Hamburg, Germany & Department of Urology, Albertinen Krankenhaus, Paul Ehrlich Strasse 1, 22763 Hamburg, Germany; dieckmannkp@gmail.com; 8 Department of Surgery, Erasmus MC-University Medical Center Rotterdam, Cancer Institute, Be-432A, PO Box 2040, 3000 CA Rotterdam, The Netherlands; h.roest@erasmusmc.nl (H.P.R.);; 9 University of Groningen, University Medical Center Groningen, Department of Medical Oncology, Hanzeplein 1, 9713 GZ Groningen, The Netherlands; j.a.gietema@umcg.nl; 10 Division of Urology, Department of Surgery, Princess Margaret Cancer Centre, University Health Network, 610 University Ave., 3-130, Toronto, ON M5G 2M9, Canada; Rob.Hamilton@uhn.ca; 11 Central Laboratory Animal Facility, Leiden University Medical Center, Einthovenweg 20, Leiden 2333 ZC, The Netherlands; d.c.f.salvatori@lumc.nl; 12 Department of Pathobiology, Anatomy and Physiology Division, Faculty of Veterinary Medicine, Utrecht University, Yalelaan 1, 3584 CL Utrecht, The Netherlands

**Keywords:** cell lines, liquid biopsies, microRNAs, mouse xenograft model, germ cell tumors

## Abstract

Liquid biopsy-based biomarkers, such as microRNAs, represent valuable tools for patient management, but often do not make it to integration in the clinic. We aim to explore issues impeding this transition, in the setting of germ cell tumors, for which novel biomarkers are needed. We describe a model for identifying and validating clinically relevant microRNAs for germ cell tumor patients, using both in vitro, in vivo (mouse model) and patient-derived data. Initial wide screening of candidate microRNAs is performed, followed by targeted profiling of potentially relevant biomarkers. We demonstrate the relevance of appropriate (negative) controls, experimental conditions (proliferation), and issues related to sample origin (serum, plasma, cerebral spinal fluid) and pre-analytical variables (hemolysis, contaminants, temperature), all of which could interfere with liquid biopsy-based studies and their conclusions. Finally, we show the value of our identification model in a specific scenario, contradicting the presumed role of miR-375 as marker of teratoma histology in liquid biopsy setting. Our findings indicate other putative microRNAs (miR-885-5p, miR-448 and miR-197-3p) fulfilling this clinical need. The identification model is informative to identify the best candidate microRNAs to pursue in a clinical setting.

## 1. Introduction

Over the past years we have witnessed a substantial increase in the number of publications focusing on liquid biopsies. These are particularly useful in the context of cancer, as non-invasive means of diagnosis and follow-up [1]. MicroRNAs are among the various liquid biopsy-based molecular biomarkers showing promise in this field. They are involved in the post-transcriptional regulation of the functionality of genes, and are crucial modulators of several biological processes, including embryonic and germ cell development [2]. One of the advantages of microRNAs as liquid biopsy-based biomarkers relates to their relative stability in body fluids. Moreover, they can be easily detected and quantified in a cost-beneficial manner, with high sensitivity and specificity [3].

Germ cell tumors (GCTs) are very diverse, comprising various histological subtypes (the most common being seminomas [SEs] and the several non-seminoma [NS] subtypes), anatomical distributions (both gonadal—testicular and ovarian—and extragonadal tumors, along the midline of the body) and afflicting a wide range of age groups (pediatric, young-adults and even old-adults) [4]. Their most fascinating characteristic is that they are developmental cancers: each tumor entity resembles a phase of embryonic and germ cell development and recapitulates the epigenetic pattern of the respective originating cell [5,6]. The main variant based on epidemiological characteristics are the so-called Type II testicular germ cell tumors (TGCTs), also known as germ cell neoplasia in situ (GCNIS)-related GCTs of the testis. They are the most common neoplasms among young-adult men in Western civilization, but are also amongst the most curable solid cancers, which could make one assume there is not much more to improve in this field [7]. However, precisely because of this, both patients and clinicians face novel unexpected challenges, including risk of overtreatment, exposing patients to unnecessary long-term side effects of chemo- and radiotherapy; and also insecurity about stratification of patients to different follow-up and treatment protocols [8,9,10,11]. The existing biomarkers used nowadays in the clinic (alpha fetoprotein [AFP], human chorionic gonadotropin subunit β [β-HCG] and lactate dehydrogenase [LDH]) are informative, but show limited utility in daily practice to respond to all these issues; therefore, better biomarkers for the disease are needed [12,13,14].

From the biological perspective over these neoplasms, microRNAs emerge as promising biomarkers [15]. In fact, a set of (embryonic) microRNAs (including the miR-371/373 cluster and the miR-367) have been pinpointed by miR-array to be actively involved in (T)GCT biology [16,17,18], and have proved their value in the past years as biomarkers of (T)GCTs in a multitude of studies with various designs, focusing mainly on type II TGCTs, but also extending to type I pediatric and extragonadal tumors [17,18,19,20,21,22,23,24,25,26,27,28,29,30]. Initial studies consisted mainly of reports of patients where microRNA determination was pursued [30] and proof-of-principle works with limited number of subjects included [31,32]. Given the promising results, larger studies were conducted, retrospective and more recently also prospective, and aimed at solving relevant clinical questions in the field [22,33]. In these works, miR-371a-3p was demonstrated to be the most remarkable biomarker [34,35,36], outperforming classical serum markers in their ability to diagnose, follow-up and predict residual disease after chemotherapy in these patients, with sensitivity and specificity over 85–90% [27,37,38]. This microRNA is related to all different histological elements of (T)GCTs, except mature teratoma, for which a proven informative biomarker is lacking so far. This specific microRNA profile in (T)GCTs might also be exploited for treatment purposes, targeting overexpressed oncogenic microRNAs and/or replenishing underexpressed tumor suppressor microRNAs [39], or even to be used for suicide gene activation [40].

For any biomarker to be introduced in the clinic, appropriate technical issues should be considered. Novel methodologies may be of use in the future, such as digital droplet PCR and next generation sequencing, which could overcome eventual unspecificity of the assays used, however, the RT-qPCR pipeline validated thus far is attractive for implementation in the clinic, since it represents a relatively low-cost and fast method for testing several patient samples in time, providing clinicians with valuable information. A recent health economic analysis estimated that miR-371a-3p could reduce the costs with germ cell tumor patients follow-up strategy by as much as 44%, especially at the expense of reducing the amount of necessary imaging in microRNA-negative cases [41]. The value of this standardized pipeline relies on appropriate quality control and normalization [23,29]. However, some important issues remain, such as hemolysis content in blood samples, as it can interfere with the detection levels of certain microRNAs [42,43], as well as the choice of analysis of serum or plasma. However, the real impact of these matters on specific assays and the best way to approach them is still debatable.

MicroRNAs can be secreted from tumor cells in various ways [44]. In spite of the data on the putative impact in the clinical setting, microRNA synthesis and secretion dynamics in (T)GCTs are still largely unknown, and a proper characterization of these processes in (T)GCTs (i.e., possible selectivity—Supplementary Figure S1—and involvement of vesicles) has not yet been tackled. Since these representative models reflect to some extent the biology of these tumors, complementing such in vitro data with further data derived from in vivo pre-clinical models could be extremely valuable to identify the most informative microRNAs for clinical application.

In a recent integrated analysis, Shen et al. [45] suggested that miR-375 is overexpressed in tissue samples from teratoma and yolk sac tumor (and mixed tumors containing these subtypes); this finding could be extremely useful, particularly in the context of primary diagnosis in cases of pediatric GCT, as well as in the metastatic context after chemotherapy in both pediatric and adult patients. The reason is that detection of (residual) mature teratoma is clinically important and challenging, being the only subtype remaining undetected by the promising miR-371a-3p. However, this data lacks validation in liquid biopsy samples so far.

The aim of this work is to investigate in detail the dynamics of microRNA synthesis and secretion in (T)GCT cell lines, correlating with patterns observed in mouse models, achieving a reliable combined in vitro and in vivo model for identifying the most promising candidate microRNAs. In addition, the impact of pre-analytical variables (hemolysis, choosing serum vs. plasma) on microRNA quantification is investigated. Moreover, the potential role of miR-375 in a liquid biopsy setting, confirming or disproving preliminary data reported on tumor tissues, is performed. This setup will be informative for other disease processes as well. 

## 2. Materials and Methods

### 2.1. Ethics Approval

Use of patient samples remaining after diagnosis was approved for research by the Medical Ethical Committee of the EMC (the Netherlands), permit no. 02.981. This included permission to use the secondary samples without further consent. Samples were used according to the ‘‘Code for Proper Secondary Use of Human Tissue in The Netherlands’’ developed by the Dutch Federation of Medical Scientific Societies (FMWV, version, 2002; update 2011). Animal experiments were approved by the Dutch Central Commission for Animal experimentation (Centrale Commissie voor Dierproeven). The study was conducted in accordance with the Declaration of Helsinki.

### 2.2. Statistical Analyses

Detailed statistical analyses performed are described under each section (see below). Data was tabulated using Microsoft Excel 2016 (Microsoft, Redmond, WA, USA) and analyzed using GraphPad Prism 6 (GraphPad Software, San Diego, CA, USA) and IBM SPSS Statistics version 24 (SPSS Inc, Chicago, IL, USA). Heatmaps of microRNA data were generated in R using the “pheatmap” clustering software package, using default settings. Venn diagrams were designed using Interactive Venn [46]. Statistical significance was set at *p* < 0.05.

### 2.3. MicroRNA Isolation, Quantification and Quality Control

For liquid biopsy-based studies (including conditioned media), microRNAs were isolated (from 50 µL samples) by the ampTSmiR test (magnetic bead-based isolation) using the KingFisher Flex System (ThermoFisher, Waltham, MA, USA), followed by cDNA synthesis, pre-amplification step (12 cycles) and real-time quantitative polymerase chain reaction (RT-qPCR), of which the pipeline has been extensively reported by us before [20,23]. A non-human microRNA spike-in (ath-miR-159a) was added in a fixed amount to the samples (2μL of a 1 nM stock solution) for quality control of RNA isolation and cDNA synthesis. All samples included in the study (except those used specifically for exploring the hemolysis effect—see below) were visually inspected for hemolysis, and none with obvious pink discoloration was used. Experiments on patient samples were done in single (sample availability issues) and in vitro/in vivo studies in duplicate, and no samples had to be excluded due to poor microRNA recovery, based on recovery of the spike-in ath-miR-159a (variation in Ct values within ± 2 Ct after pre-amplification). Ct values were normalized to the endogenous reference miR-30b-5p. MicroRNA levels were relatively quantified according to the 2^−ΔΔCT^ method (after normalization to housekeeping miR-30b-5p and to the average ΔCt of the control/normal male samples included) and plotted in log2 format for readability. To assure quality control, RT-qPCR efficiency and inter-plate comparability, serial dilutions (1:8) of cDNA from SE-like cell line TCam-2 [47] were included for each assay tested. A no template control was included for every assay in the cDNA synthesis, pre-amplification steps and RT-qPCR. RT-qPCR was run in QuantStudio 12K Flex Real-Time PCR System (ThermoFisher Waltham, MA, USA).

### 2.4. MicroRNA Profiling

For all four cell lines (TCam-2, NCCIT, NT2 and 2102Ep, see below), matched conditioned media, fetal calf serum, mouse xenografts, sera/plasma samples and cerebral spinal fluid (CSF) samples, microRNA profiling was performed on bead-captured microRNAs (as described above). Samples were reverse transcribed using Megaplex Primer Pool A and B, followed by a pre-amplification step of 12 cycles (using Megaplex PreAmp Primer Pool and TaqMan PreAmp Master Mix, ThermoFisher, Waltham, MA, USA). The product was loaded on the matching TaqMan Low-Density Array (TLDA) Cards A+B. All reagents were purchased from Thermo Fisher/Life Technologies (ThermoFisher, Waltham, MA, USA). For the CSF samples only card A was run; individuals had the following age and gender: 44, male; 43, male; 42, male; and 54, female. TaqMan microRNA array output data (sds files) were uploaded in the ThermoFisher Cloud App (https://www.thermofisher.com/mysso/loginDisplay) and analyzed using defined threshold settings for each individual microRNA. Cq values were exported and filtered for poor amplification performance; for consistency we will use Ct when discussing filtered Cq values.

To determine whether the microRNA isolation method could impact on our results throughout the experiments and several datasets, TLDA cards using cDNA obtained from total RNA extraction were compared to TLDA cards using cDNA obtained after microRNA bead-capture, for each of the four cell lines. Additionally, to determine the effects of pre-amplification on comparisons between cells and matched media, the Ct values from the TLDA cards for the 2102Ep cell line with and without pre-amplification step were compared. 

### 2.5. Cell Lines

Cell lines were cultured as previously described; for details on these cell lines please refer to [48]. In brief, TCam-2, NT2 and 2102EP were cultured in RPMI 1640 medium with glutamax, and NCCIT in DMEM (high glucose) glutamax, in both cases with 10% fetal calf serum (HyClone, Perbio, UT, USA). In all experiments, fetal calf serum was used as a negative control. The identification of the cell lines used was determined before based on genome wide copy number variations [47]. 

To determine whether the amount of the miR-371/373 cluster and miR-367 is balanced between cells and matched media in each cell line, microRNA profiling using TLDA cards was performed and waterfall plots were built using the raw Ct values of the cells. The same procedure was used for the respective media, using the order of Ct values of cells as reference. Finally, ∆Ct was calculated.

To further investigate the stability of the secretion process and how active secretion could be affected by several stressing (metabolic) conditions, miR-371a-3p levels were assessed in medium of TCam-2 and NCCIT cells with different proliferation rate (cells grown over 192 h, with medium sampling and cell counting over several time points) and with different conditioned medium incubation temperatures (room temperature for 27 h; on ice for 27 h; incubated at 37 °C with 5% CO2; frozen and thawed five times and ten times). For quantification, both regular cell count (confluent, in a Bürker counting chamber) and EVQuant methodology (extracellular vesicles [EVs] per mL, a technology recently developed by the Erasmus MC Rotterdam as a novel, practical and low-cost approach for EVs quantification) were used [49].

To test whether the microRNAs are packed into exosomes, 50 µL conditioned media of TCam-2 cells was subjected to four experimental conditions: direct microRNA bead-based capture, as described above (AmiR); and total exosome isolation (using Total Exosome Isolation Reagent, ThermoFisher) to reach a pre-enriched exosome suspension, followed by either microRNA bead-based capture (Ex AmiR), Total Exosome RNA & Protein Isolation Kit (Ex Kit, ThermoFisher, Waltham, MA, USA) or immunoprecipitation with superparamagnetic beads coated with CD63 antibodies (Ex 63, Exosome—Dynabeads^®^ Human CD63 Isolation/Detection, ThermoFisher, Waltham, MA, USA). For quality control of microRNA purification and cDNA synthesis, the non-human miRNA spike-in ath-miR-159a was used (added during all microRNA purifications in a standard concentration of 0.2 μL per sample from a 1 nM stock solution). Dependent on the method of microRNA purification, the spike-in was added to lysis buffer (ABC beads) or to 1 × PBS (Exosome RNA isolation). To determine the efficiency of exosome isolation, C. elegans microRNA cel-miR-39-3p was used (added to cell medium prior to exosome isolation, in a standard concentration of 0.2 μL per sample from a 1 nM stock solution). Spike-ins, hsa-miR-30b-5p and hsa-371a-3p were quantified in all four situations using RT-qPCR as described above. Non-detection of cel-miR-39-3p in quantitative analysis was used as proof of successful exosome isolation. The outline of this experiment is illustrated in Supplementary Figure S2.

### 2.6. Mouse Model

In order to extend the reach of our microRNA-identification model, we used a mouse xenograft model already described by us (which contains both benign and malignant teratoma samples as determined by teratoma assay); for details about the origin of animals and related information please refer to [50]. Briefly, the aforementioned (T)GCT cell lines and also human pluripotent stem cells (hPSCs) and induced pluripotent stem cells (IPS) were injected subcutaneously into immunodeficient mice and tumor xenografts grew until a maximum size of 2cm^3^ (endpoint), after which they were collected for histological evaluation and microRNA isolation. Endpoint mouse EDTA plasma samples were also obtained (Supplementary Figure S3). As negative controls, a mixture of normal mouse tissues (*n* = 2) and plasma from normal mice (*n* = 3) were used. The microRNA isolation using magnetic beads and TLDA-based microRNA profiling was subsequently performed in all samples as previously described [51]. Relevant microRNAs were then validated by targeted analyses as previously stated. The performance of these microRNAs in discriminating teratoma from control patients was assessed through receiving operating characteristic (ROC) curve construction. Youden’s method [52,53] was used to achieve a cut-off to maximize the sensitivity and specificity. In addition, area under the curve (AUC), sensitivity, specificity, positive predictive value (PPV), negative predictive value (NPV) and accuracy were ascertained.

### 2.7. Hemolysis Analyses and Heparin Contamination

The “miR-23a/451a ratio”, reported by Shah et al. [42] as an accurate measure of hemolysis in serum samples, has not been validated in the same experimental conditions as ours (i.e., after bead-based microRNA capture followed by cDNA synthesis and pre-amplification of the purified microRNAs), hence the same cutoffs described by the authors may not apply to our work. So, we set out to explore and validate another more appropriate methodology of assessing this issue in our experimental conditions. A total of 775 serum samples (follow-up samples from TGCT patients provided by University Medical Center Groningen (UMCG) reported recently [37] and pooled blood bank-derived sera provided by Sanquin, Amsterdam, The Netherlands) were included and hemolysis was scored from 0 to 5, based on visual inspection (pink discoloration), as described before [42]. After bead-based microRNA capture, cDNA synthesis and pre-amplification of the product, RT-qPCR for hsa-miR-23a-3p, mmu-miR-451a, ath-miR-159a, hsa-miR-30b-5p and hsa-miR-372-3p was performed, and the miR-23a/451a ratio was calculated. ROC curve analysis assessed the performance of the ratio in discriminating hemolysis presence, and Youden’s index [52] was used to achieve the optimal cutoff. Mann-Whitney U-test and Kruskal-Wallis test were employed as appropriate for assessing differences among score groups.

For exploring the impact of heparin contamination in microRNA quantification, a subset of graft preservation fluids (*n* = 8) from patients undergoing kidney transplantation (described by us in [54]) that were shown to be contaminated with heparin (*n* = 4) was selected, and subsequently quantified after microRNA isolation using miRNEAsy spin columns or after bead capture followed by pre-amplification. Ct values were determined and compared with or without heparinase 1 treatment.

### 2.8. Serum vs. Plasma Analyses

Fifty pairs of matched serum and EDTA plasma samples from normal male blood donors (controls) were included in the study (obtained from Sanquin, Amsterdam, the Netherlands). After microRNA isolation (performed as described above), RT-qPCR for ath-miR-159a, hsa-miR-30b-5p and hsa-miR-371a-3p was performed. Moreover, an additional set of 11 pairs of serum/plasma samples were included, for which hsa-miR-23a-3p, mmu-miR-451a, ath-miR-159a, hsa-miR-30b-5p, hsa-miR-371a-3p and hsa-miR-375 were also profiled. The Wilcoxon matched-pairs signed rank test was used for assessing differences among Ct values of specific targets between serum/plasma samples of matched individuals.

### 2.9. MicroRNAs Decrease after Orchiectomy

A cohort of 12 clinical stage I TGCT patients (selected from a previous work [25]) and five normal male blood donors (controls) was included in the study. The microRNA isolation and RT-qPCR for ath-miR-159a, hsa-miR-30b-5p, hsa-miR-371a-3p, hsa-miR-372-3p, hsa-miR-373-3p, hsa-miR-367 and hsa-miR-375 were performed. Inter-group differences were compared using the Mann-Whitney U test or Kruskal Wallis tests, as appropriate. For paired variables (microRNA levels along different time points) the Friedman test was employed. Dunn’s test for multiple comparisons was used and all reported p-values are two-tailed and adjusted to this. Spearman’s correlation test was used to correlate two continuous variables. In patients with multiple samples within 24h after orchiectomy, data over time was plotted as percent of the preoperative microRNA levels, and serum half-life was estimated.

### 2.10. MicroRNA-375 Analyses

In order to confirm or disprove the proposed value of miR-375 as a biomarker of GCTs, namely related to the teratoma and yolk sac tumor histological subtypes [45], a cohort of 113 serum samples from 36 patients undergoing chemotherapy followed by retroperitoneal lymph-node dissection (RPLND) was selected (from a previous cohort already reported by us [24]), and additionally 12 normal male blood donors were included. Samples were collected in three time points: pre-chemotherapy; post-chemotherapy and pre-RPLND; and post-RPLND. An illustration of the rationale and workflow is presented in Supplementary Figure S4. Additionally, a second cohort of sera samples from 26 patients in pediatric/young-adult age range (including both type I and type II tumors with teratoma and yolk sac histologies plus one ovarian dysgerminoma) and 10 sera samples from pediatric individuals with no neoplastic conditions and within the same age range were included. RT-qPCR for both the targets hsa-miR-371a-3p and hsa-miR-375 was performed as previously described and the same quality control measures were pursued. Statistical analysis was performed as detailed in the above section.

## 3. Results

### 3.1. MicroRNA in vitro Dynamics

#### 3.1.1. Various microRNAs are Specifically Secreted by the Different (T)GCT Cell Lines

Firstly, to investigate the potential impact of the methodology applied on microRNA isolation (after exclusion of inappropriate curves and cases with no evidence of microRNAs in cells) we investigated the results after either bead-based captured microRNAs or non-bead-based extraction from total RNA. The results show very minor changes in Ct values among the matched samples using the two methods, demonstrating that the bead-capture process is not saturated, so comparisons between the various datasets are informative (Supplementary Figure S5A). Also, the variability among samples with and without the pre-amplification step was minor, with a median of 10.25 Ct difference among samples (Supplementary Figure S5B), so that comparisons are not influenced by the pre-amplification step used.

**Figure 1:**
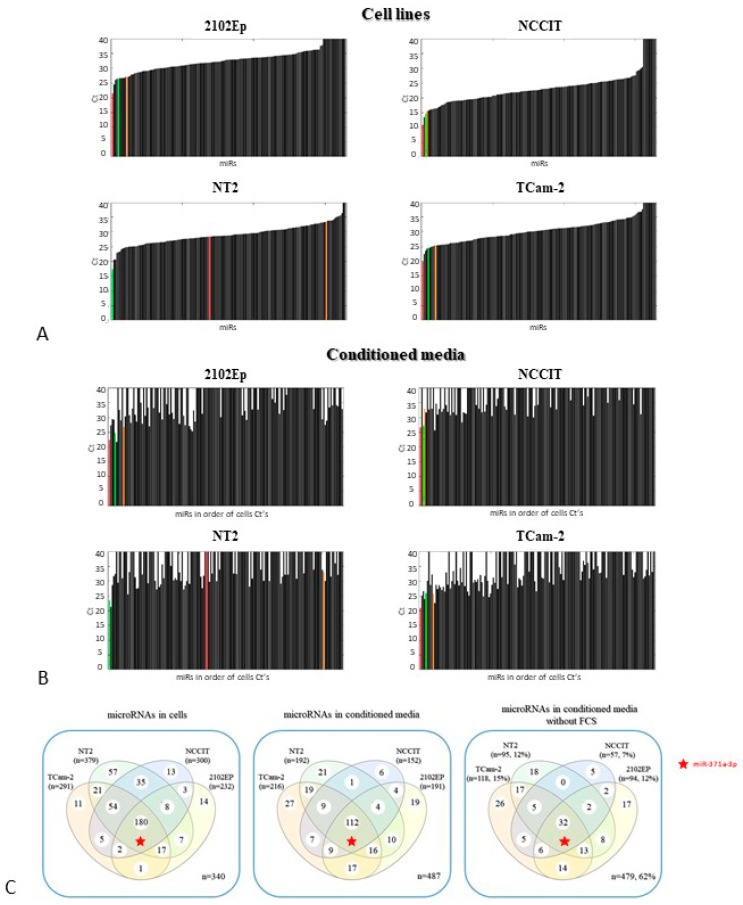
microRNA expression levels in cell lines and matched conditioned media. (**A**,**B**) Waterfall plots showing Ct values for microRNAs in cell lines (**A**) and matched conditioned media (**B**) (2102Ep, NCCIT, NT2 and TCam-2). Targets miR-371a-3p, miR-372-3p and miR-367 are highlighted in color (orange, red and green, respectively). Each cell line/media underwent two TLDA evaluations, one for card A and one for card B; (**C**) Venn diagram showing detected microRNAs in cells (left panel) and matched conditioned media (middle panel), and microRNAs specifically secreted by each cell line after eliminating the microRNAs already present in the fetal calf serum (i.e., negative control, right panel). Target miR-371a-3p is highlighted by a star. Numbers outside the venn diagram (not included in any ellipse) refer to microRNAs not detected in any sample. Abbreviations: FCS: fetal calf serum.

We then first included all profiled microRNAs and looked specifically at the patterns of expression of the panel known to be relevant in GCTs (miR-371/373 cluster and miR-367) based on multiple independent studies (see Discussion section for further information). The TLDA data was analyzed and the targets miR-371a-3p, miR-372-3p and miR-367 were specifically highlighted in color (Figure 1A–B). These three microRNAs were indeed among the highest expressed in TCam-2, 2102EP and NCCIT (both in cells and respective media). Specifically, for NT2, only miR-367 was amongst the highest expressed (again both in cells and respective media).

Out of the 768 microRNAs profiled, 477, 389, 468 and 536 were not detected in TCam-2, NT2, NCCIT and 2102EP cell lines, respectively. Only 180 microRNAs were detected in all four cell lines. A similar pattern was found for the matched media; 552, 576, 616 and 577 microRNAs were not in the detection range for the TCam-2, NT2, NCCIT and 2102EP media, respectively, and only 112 were detected in all four media (Figure 1C, left and middle panel).

Hence, we then focused only on microRNAs in the detection range (Ct values <34) for each cell line. Their distribution in cell lines is depicted in Supplementary Figure S6, along with their distribution in matched media and the delta Ct (∆Ct) values (using the order of the cell lines as reference). There is not a direct proportional association between microRNA amounts/content in matched cells and media, meaning that some microRNAs are indeed selectively secreted. 

In order to identify microRNAs specifically secreted by each cell line, analysis of the fetal calf serum (negative control) was also taken into account. Indeed, the number of microRNAs detectable in conditioned media of the several cell lines is substantially lower after exclusion of those already present in the fetal calf serum (Figure 1C, right panel), resulting from non-human specificity of the assays used. In the end, only 15%, 12%, 7% and 12% of the 768 profiled microRNAs fulfill these criteria for TCam-2, NT2, NCCIT and 2102Ep, respectively.

In line with that, considering the microRNAs detectable in cells, 101/291 (34.7%), 81/379 (21.4%), 48/300 (16.0%) and 67/232 (28.9%) microRNAs were found to be specifically secreted by TCam-2, NT2, NCCIT and 2102EP cell lines, respectively (Ct <34 in media and >34 in fetal calf serum). Likewise, 125/291 (43.0%), 213/379 (57.0%), 182/300 (60.7%) and 103/232 (44.4%) microRNAs were demonstrated not to be secreted into conditioned media of TCam-2, NT2, NCCIT and 2102EP, respectively (Ct > 34 in media and in fetal calf serum). Importantly, in most of the cases, the miR-371/373 cluster and miR-367 were found to be present in the final list of specifically secreted microRNAs (Table 1; raw data available in Supplementary File 1). 

#### 3.1.2. Secretion of miR-371a-3p is Minimally Influenced by Cell Count

**Figure 2:**
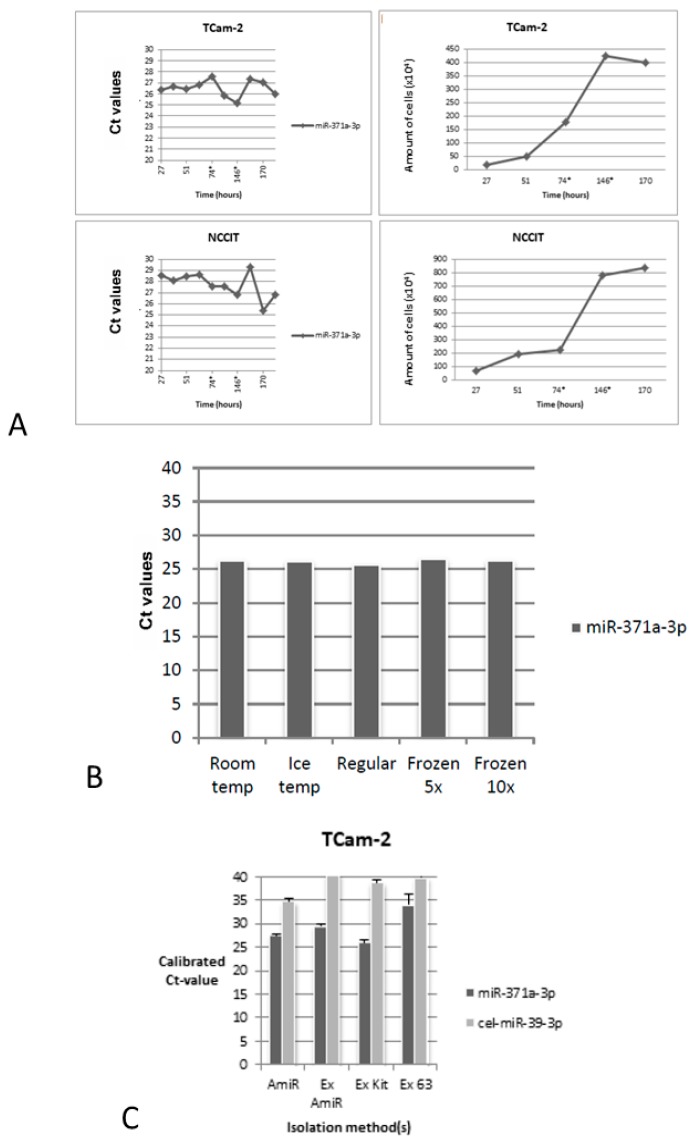
Effect of cell proliferation, multiple freezing/thawing and exosomal isolation in microRNAs detection in cell media. (**A**) Cell proliferation (right) and effect on miR-371a-3p Ct values for TCam-2 and NCCIT media. The asterisk indicates the time of refreshment of medium; (**B**) miR-371a-3p Ct values for conditioned medium of TCam-2 according to different incubation temperatures and freezing/thawing; (**C**) miR-371a-3p Ct values in TCam-2 cell medium after microRNA bead-based isolation or after exosomal fraction isolation by different methods. Absence of cel-miR-39-3p supports that exosome isolation step was successful. High Ct values for cel-miR-39-3p were depicted in bead isolation (but still lower than in exosome isolation) because of low concentration of the microRNA. Abbreviations: AmiR—A-beads microRNA isolation; Ex AmiR—total exosome isolation prior to A-beads microRNA isolation; Ex Kit—total exosome isolation prior to RNA isolation with total exosome RNA isolation kit; Ex 63—exosome isolation using Dynabeads coated with anti-CD63 antibodies.

Levels of miR-371a-3p were assessed in TCam-2 and NCCIT conditioned medium when cells were at various phases of culture density. The Ct values of this microRNA remained stable in TCam-2 and NCCIT with different proliferation indexes, with only slight fluctuations upon medium change (at 74 and/or 146 h). Ct values after 192 h of culturing were the same (for TCam-2) and only slightly lower (for NCCIT) when compared to the first time point (27 h), despite a continuous growing activity of cells in culture (Figure 2A). This suggests secretion is not/minimally influenced by cell count. Furthermore, when comparing both TCam-2 and NCCIT cell lines, the amount of extracellular vesicles as determined by EVQuant methodology influences more the miR-371a-3p Ct values than the amount of cells; despite NCCIT showing greater amount of cells in the considered timepoint, the number of EVs is lower, rendering higher Ct values for miR-371a-3p. The opposite scenario is observed for TCam-2 (Table 2). Also, lowering incubation temperature or even multiple freezing/thawing did not influence miR-371a-3p levels in TCam-2 conditioned medium, underscoring the stability of the microRNA after the secretion process (Figure 2B).

#### 3.1.3. Secretion of miR-371a-3p by TCam-2 Cells Seems to Occur via Exosomes

Results of the experiments evaluating whether the specific microRNAs are placed into exosomes are summarized in Figure 2C. Calibrator ath-miR-159a and normalizer hsa-miR-30b-5p were similar in all fractions, as expected. Exosomal isolation from TCam-2 medium succeeded, proven by undetectable cel-miR-39-3p for all exosome isolation methods. The calibrated Ct-value for miR-371a-3p after total microRNA isolation (AmiR) was similar to the Ct-value after exosomal miRNA isolation using the Total Exosome RNA and Protein Isolation Kit (Ex Kit) and to a lesser extent after using the paramagnetic beads (Ex AmiR). Secretion of miR-371a-3p in TCam-2 is therefore predominantly related to the exosomal fraction. After immunoprecipitation with CD63+ Dynabeads^®^ (Ex 63), the Ct-value for miR-371a-3p was higher, indicating a selection of the total fraction of exosomes using this method.

### 3.2. MicroRNAs as Biomarkers using an in vivo Mouse Model

#### 3.2.1. MicroRNA Profiling Allows for Identification of Potential Candidates

Using a similar methodology as in the in vitro analyses, normal mice samples (both tissue mixtures and plasma) were used as negative controls. A total of 173 and 176 microRNAs were in the detection range in the two normal mouse tissue samples (157 shared by both), and hence were discarded from the analysis. In plasma samples, a total of 231, 220 and 230 microRNAs were detected in the normal mouse plasma samples (190 shared by all three), and were also discarded from the analysis.

**Figure 3:**
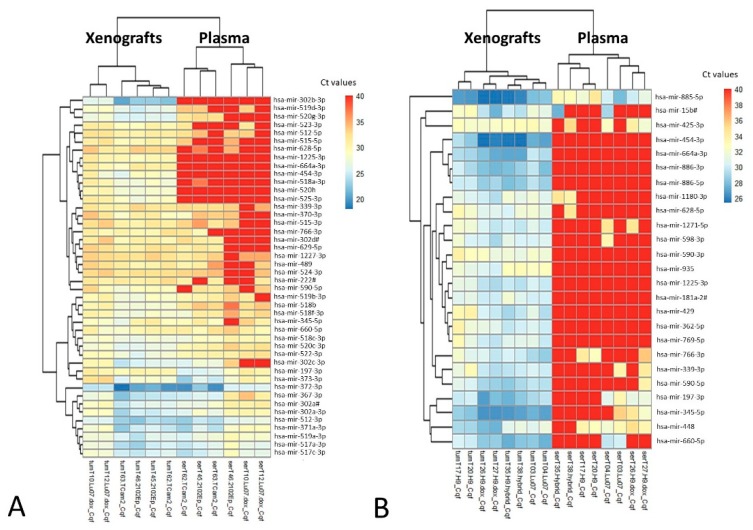
Prediction of tumor specific microRNAs able to be detected in liquid biopsy samples. MicroRNA expression in mice tumor xenografts vs. matched endpoint plasma samples. Ct values for mouse human tumor xenografts with malignant (**A**) and benign (**B**) histology vs. matched endpoint plasma samples from the same mice. Samples on the left annotated as “tum” refer to xenografts, while those on the right annotated with “ser” refer to plasma samples. Notice that in (**A**) the microRNAs already shown to be informative (cluster 371/373 and microRNA-367) are detected both in xenografts and plasma samples.

Then we aimed at uncovering the human microRNAs that were able to be identified specifically in the liquid biopsy context. We focused on the mouse human tumor xenografts with malignant histology (derived from injection with Lu07dox, TCam-2 and 2102Ep cells), representing the human GCTs closely. Among the microRNAs consistently detected in all these xenografts (*n* = 44), a cluster of microRNAs are found to be specifically detectable in the matched endpoint plasma samples; among these we find the miR-371/373 cluster and miR-367 (Figure 3A). Additionally, we studied the xenografts with benign histology (i.e., teratoma, derived from injection with H9, H9dox, H9hybrid and Lu07). Only 25 microRNAs were consistently detected in all these xenografts and their detection in matched endpoint plasma samples was infrequent. However, the miR-885-5p was detected in all matched plasmas, and also miR-448 and miR-197-3p were detected in at least 50% of plasma samples, making them candidates for detecting mature/benign teratoma in liquid biopsies (Figure 3B). Focusing on cell lines and respective conditioned media, miR-885-5p was detected in NT2 cells and respective medium, while miR-448 was found in NCCIT cells only and not in the conditioned medium.

#### 3.2.2. MicroRNA Targeted Analyses of Potential Candidates Confirm the Value of the in vivo Model

Given our data on microRNA profiling indicating miR-885-5p, miR-448 and miR-197-3p as possible candidate biomarkers of (benign) mature teratoma, and given the relevance of discovering such a microRNA for the field, these microRNAs were selected for further validation by targeted analyses, using a cohort of post-chemotherapy (but pre-RPLND) serum samples (clinicopathological descriptions are available in Supplementary Table S1) and six normal male sera.

**Figure 4:**
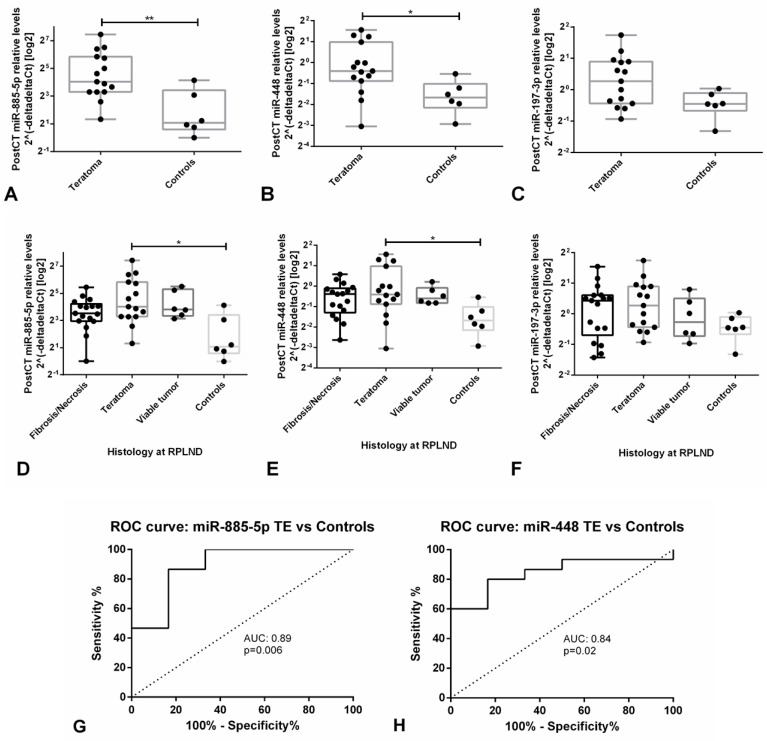
Validation of candidate microRNAs for teratoma histology in a post-chemotherapy retroperitoneal lymph node dissection series. (**A**–**C**) Relative levels of miR-885-5p, miR-448 and miR-197-3p in serum of patients with teratomas and healthy males; (**D**–**F**) Relative levels of miR-885-5p, miR-448 and miR-197-3p in sera of patients with the various histological tumor types and healthy males; (**G**,**H**) ROC curves for the discrimination among teratoma patients and healthy male individuals. Because controls were included in the comparison, the reference sample was the one showing the highest Ct value in these analyses. Abbreviations: AUC—area under the curve; CT—chemotherapy; ROC—receiving operator characteristic; RPLND—retroperitoneal lymph node dissection; TE—teratoma.

Indeed, relative serum levels of both miR-885-5p and miR-448 were significantly higher in teratoma patients when compared to healthy males (*p* = 0.0046 and *p* = 0.0140, respectively), even after adjusting for multiple comparisons (adjusted p-values of 0.0176 and 0.0291, respectively). The same tendency was observed for miR-197-3p, although not reaching statistical significance (*p* = 0.0763) (Figure 4A–F). Relative levels of miR-885-5p and miR-448 allowed for discrimination of teratoma from control patients with an AUC of 0.89 and 0.84, depicting good performance parameters, further demonstrating the value of our model (Figure 4G,H).

Moreover, when defining “a positive test” when at least one of these two microRNAs is above the defined cutoff, discrimination performance increases, showing sensitivity of 93.3% and accuracy of 90.5%. Performance parameters of these two microRNAs are depicted in Table 3. 

The relative levels of these microRNAs were, however, not significantly different among patients with only fibrosis/necrosis, teratoma and with viable tumor (non-teratoma) histological compositions, limiting their value in this specific clinical setting (Figure 4D–F).

### 3.3. Validation and Proof of Concept: miR-371a-3p and miR-375 as Candidate Biomarkers of Germ Cell Tumors in Liquid Biopsy Setting 

#### 3.3.1. miR-371a-3p Levels Continuously Decline after Orchiectomy and Half-Life is Short

**Figure 5:**
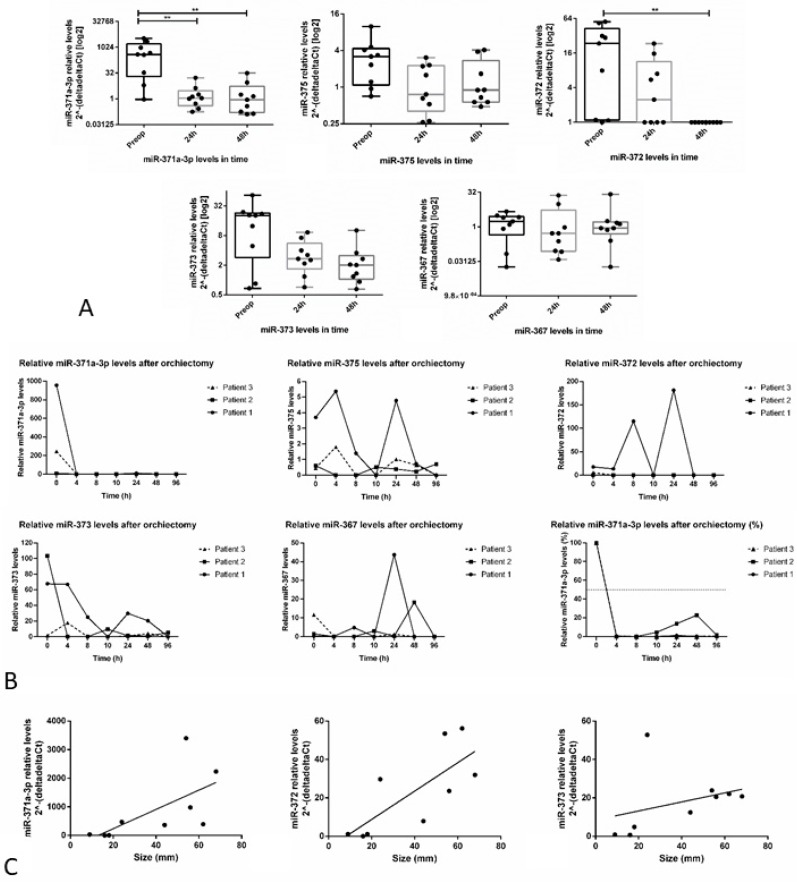
Relative microRNA expression levels after orchiectomy. (**A**) Box plots showing relative miR-371a-3p, miR-375, miR-372-3p, miR-373-3p and miR-367 expression levels in serum samples of clinical stage I testicular germ cell tumor patients (*n* = 9) at different time points after orchiectomy. (**B**) Line graphs showing relative miR-371a-3p, miR-375, miR-372-3p, miR-373-3p and miR-367 levels in 3 patients with multiple serum samples collected within the first 24 h after orchiectomy. The bottom right graph represents the percent of decrease of miR-371a-3p over time (and the dashed line depicts the 50% reduction level); (**C**) Scatter plots showing the correlation between relative miR-371a-3p, miR-372-3p and miR-373-3p levels and primary tumor size in patients with clinical stage I testicular germ cell tumor (*n* = 9).

A complete description of clinicopathological features of the cohort is depicted in Supplementary Table S2. There was a significant decrease of relative levels of miR-371a-3p and miR-372-3p after orchiectomy, seen at 48h for miR-372-3p (*p* = 0.0029) and already at 24h for miR-371a-3p (*p* = 0.0066 for 24h and *p* = 0.0029 for 48h after orchiectomy, respectively). An apparent continuous decrease is also seen for miR-373-3p, although it did not reach statistical significance. No significant differences were observed for miR-367 nor miR-375 (Figure 5A). For the three patients with multiple samples collected within 24h after the orchiectomy, a continuous steady decrease of microRNA relative levels was clear only for miR-371a-3p, with the remaining microRNAs showing fluctuations in expression levels over time. The half-life of miR-371a-3p in these patients was <4h (Figure 5B).

Fluctuations in some targets (namely miR-372-3p and miR-367-3p) may be explained by some unspecificity of the assays used. To justify this, we have performed cDNA synthesis (using the TCam-2 cell line) by pooling the RT-primers for miR-371a-3p, miR-372-3p, miR-373-3p and miR-367-3p together, plus four extra pools, excluding one of the RT-primers for the mentioned assays in each pool. Our results demonstrate that when leaving out miR-371a-3p RT-primer, no amplification product is depicted in the PCR reaction, both with and without pre-amplification, assuring the specificity of the assay. The exact same result was seen with miR-373-3p.

However, for the pool that excluded miR-372-3p, amplification of this target was seen both with and without pre-amplification step (Ct values of 31.8 and 21.7, respectively); and in the pool leaving out miR-367-3p, amplification product was not depicted in the absence of pre-amplification, but only after pre-amplification step (Ct value of 27.7). In order to further explore the unspecificity of the assay for miR-372-3p, further RT-primer pools were made, always leaving out the miR-372-3p RT-primer and additionally excluding one more of the other targets (either miR-371a-3p, miR-373-3p, miR-367-3p, miR-30b-5p or ath-miR-159a). In all these situations a PCR reaction could detect some amplification of miR-372-3p; the lowest amount of unspecific amplification product was depicted when omitting simultaneously both miR-372-3p and miR-373-3p RT-primers (Supplementary Figure S7).

Patients’ age at diagnosis was not significantly correlated with any microRNA relative expression levels. For miR-371a-3p and miR-372-3p there was a significant, strong positive correlation between tumor size and relative expression levels (r_s_ = 0.75 and r_s_ = 0.8, *p* = 0.025 and *p* = 0.014, respectively). A similar correlation was also found for miR-373-3p relative expression levels, although it did not reach significance (r_s_ = 0.617, *p* = 0.086) (Figure 5C). miR-371a-3p relative expression levels were positively correlated with miR-372-3p and miR-373-3p levels (r_s_ = 0.817 and r_s_ = 0.800, *p* = 0.011 and *p* = 0.014, respectively). There were no significant differences between the preoperative levels of all microRNAs among SE and NS samples.

#### 3.3.2. Analysis of TLDA Data Suggests miR-371a-3p, but not miR-375, as a Specific Biomarker in Liquid Biopsy Setting

When analyzing TLDA data referring to miR-375, and comparing with the miR-371a-3p, distinct profiles are observed: while miR-371a-3p is found to be secreted into the media of all four (T)GCT cell lines, miR-375 is only detected in media of NT2 and TCam-2 (Supplementary Figure S8A,C). Also, in our mouse xenograft model dataset, miR-371a-3p is clearly detected in mouse endpoint plasma exclusively in cases of malignant histology and not in controls or benign teratoma, whereas miR-375 is detected in all situations as well as in controls (Supplementary Figure S8B,D). Also, while miR-371a-3p was not detected in any of the 16 normal male serum samples, miR-375 was detected in 11/16 of these samples.

#### 3.3.3. Validation Studies in Patient-Derived Data Confirm the Clinical Utility of miR-371a-3p, but not miR-375, as Liquid Biopsy Biomarker for GCTs (types I and II)

Post-chemotherapy miR-371a-3p relative levels were significantly higher in patients with viable tumor (non-teratoma) at RPLND when compared to those presenting with teratoma only or fibrosis/necrosis (adjusted p-values of 0.0228 and 0.0348, respectively), independently replicating our previous observations on the series [24]. However, no significant variation was observed for miR-375 relative levels. 

**Figure 6:**
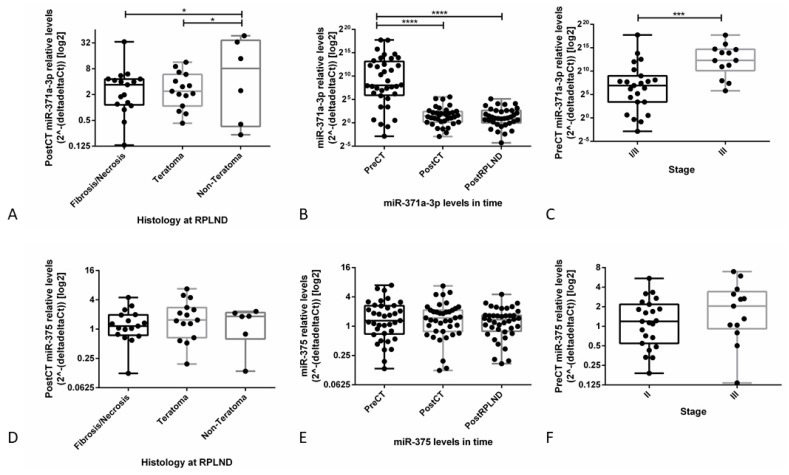
miR-375 and miR-371a-3p as serum biomarkers for TGCTs in the context of post-chemotherapy RPLND. Boxplots showing: relative post-chemotherapy miR-371a-3p (**A**) and miR-375 (**D**) expression levels in TGCT patients with different histological subtypes (fibrosis/necrosis, teratoma, viable tumor) at RPLND; relative miR-371a-3p (**B**) and miR-375 (**E**) expression levels in different time points: pre-chemotherapy, post-chemotherapy and post-RPLND; and relative pre-chemotherapy miR-371a-3p (**C**) and miR-375 (**F**) expression levels according to disease stage (I/II vs. III). Abbreviations: CT—chemotherapy; RPLND—retroperitoneal lymph node dissection.

Also, the pre-chemotherapy miR-371a-3p relative levels were significantly higher than those after chemotherapy and after RPLND (adjusted p-values <0.0001 for both), while for miR-375 no significant changes were noted. Relative levels of miR-371a-3p in the pre-chemotherapy period were associated with tumor burden, being significantly higher in stage III disease (*p* = 0.0009) (Figure 6A–C), while again no significant variation was seen for miR-375 (Figure 6D–F). Importantly, in all these three circumstances, miR-375 levels did not differ significantly from the ones observed in normal male controls (Supplementary Figure S9).

**Figure 7:**
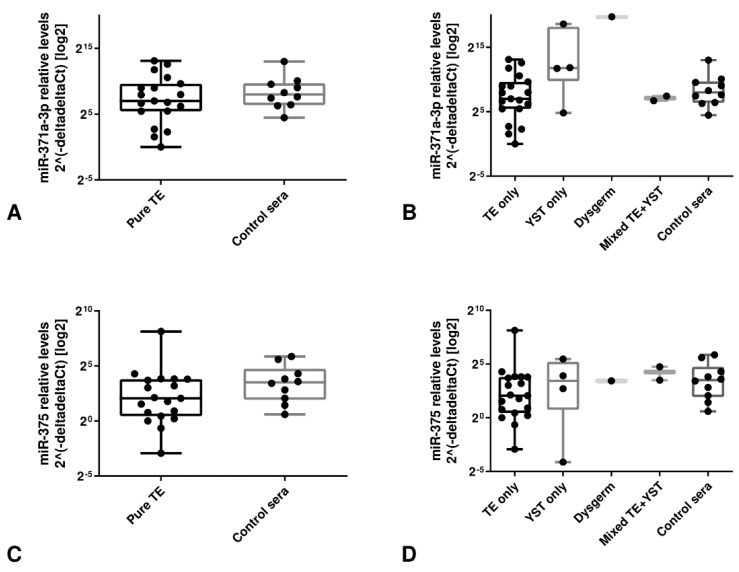
miR-375 and miR-371a-3p as serum biomarkers for GCTs with teratoma/yolk sac tumor histologies. Boxplots showing relative levels of miR-371a-3p (**A**,**B**) and miR-375 (**C**,**D**) among different histological subtypes and controls within the same age range. Because controls were included in the comparison, the reference sample was the one showing the highest Ct value in these analyses.

Additionally, pre-chemotherapy levels of miR-371a-3p significantly and positively correlated with the size of the metastatic mass, the serum β-HCG and the serum LDH before treatment (r_s_ = 0.50, *p* = 0.002; r_s_ = 0.44, *p* = 0.008; and r_s_ = 0.69, *p* < 0.001, respectively); the same tendency was found for AFP levels although it did not reach significance (r_s_ = 0.319, *p* = 0.062). None of these correlations or tendencies was found for miR-375. Both microRNAs levels did not correlate significantly with patients’ age (*p* = 0.117 and *p* = 0.207)A description of the diagnoses of each individual included in the cohort of teratoma and yolk sac tumor cases is provided in Supplementary Table S3. Confirming previous findings of the limited use of miR-371a-3p in the context of teratoma-predominant tumors [23,26], there were no significant differences between relative levels of miR-371a-3p among normal male sera and sera corresponding to patients with pure teratoma (Figure 7A). Relative levels of this microRNA were, however, higher when considering the pure yolk sac tumors and the dysgerminoma (Figure 7B). For miR-375, the relative levels were the same among all tested sera samples, including all histological subtypes and controls (Figure 7C–D).

### 3.4. Technical Considerations when Working with Liquid Biopsy-Based Biomarkers

#### 3.4.1. The effect of Hemolysis and Heparin Contamination is Absent if Bead-Based Capture is Performed

**Figure 8:**
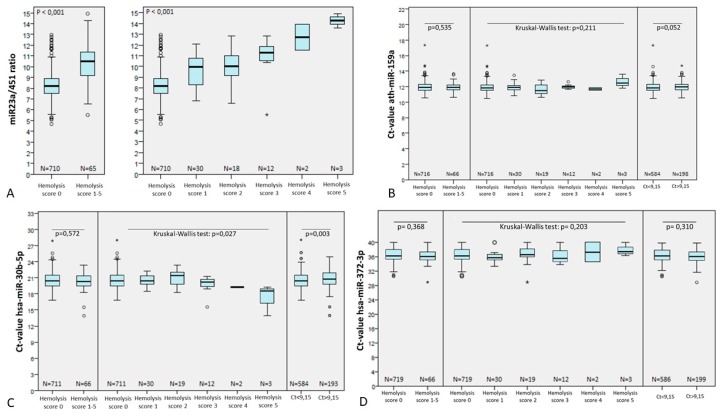
The effect of hemolysis on microRNA levels. miR-23a/451a ratio (**A**) and Ct-values of the spike-in ath-miR-159a (**B**), normalizer hsa-miR-30b-5p (**C**), and target assay hsa-miR-372a-3p (**D**) according to hemolysis visual scoring and the pre-determined miR-23a/451a ratio cutoff (9.15).

Regarding presence of hemolysis, visual inspection demonstrated a distribution of hemolysis score in the cohort under evaluation as follows: 710 samples with score 0, 30 with score 1, 18 with score 2, 12 with score 3, two with score 4, and three with score 5. The miR-23a-3p was stable in all samples, with no significant differences among cases with or without hemolysis (*p* = 0.421). Also, the miR-23a/451a ratio was significantly lower in samples with lower hemolysis scores (*p* < 0.001, Figure 8A).

By ROC curve analysis, the miR-23a/451a ratio was able to discriminate samples with no hemolysis (score 0) from those with evidence of hemolysis (scores 1–5) with an AUC of 0.812. The optimal cutoff for discriminating these groups of samples in our work was a ratio of 9.15, allowing for a sensitivity of 77% and specificity of 80% in the discrimination (Supplementary Figure S10). Samples were then categorized in respect to hemolysis presence based on this cutoff.

We then set out to assess the impact of hemolysis on specific assays, scored by the two different methods (visual inspection and our predetermined cutoff). There was no significant impact of hemolysis (determined by either method) on the microRNA isolation procedure itself, as there were no significant differences in the Ct values of spike-in ath-miR-159a (Figure 8B). There were also no significant differences in Ct values of the normalizer miR-30b-5p between samples with hemolysis scores 0 vs. scores 1–5. miR-30b-5p Ct values were significantly different among score groups and miR-23a/451a ratio groups, but the magnitude of this difference was minor and, importantly, at the expense of samples containing severe hemolysis (scores 4–5, for which Ct values were lower), as illustrated in Figure 8C. Finally, there was no significant impact of hemolysis (determined by either method) in the Ct values of the specific target assay miR-372a-3p (Figure 8D).

As a proof of concept, considering the RPLND patient cohort included in our study, visual inspection showed only samples with hemolysis scores 0–1 (Supplementary Figure S11A). Of the 144 samples, 26 (18%) showed a miR-23a/451a ratio above the defined cutoff. However, hemolysis (defined by the aforementioned cutoff) showed no significant impact on Ct values of either the spike-in ath-miR-159a, the normalizer hsa-miR-30b-5p or the target assays hsa-miR-371a-3p or hsa-miR-375 (Supplementary Figure S11B).

Regarding fluid samples containing heparin contamination (kidney graft preservation solution), as another example of possible detection interference, we demonstrate that Ct values of both spiked-in synthetic microRNAs cel-miR-54-3p and cel-miR-39-3p, as well as the endogenous microRNAs hsa-miR-21-5p and hsa-miR-505-3p, clearly are not affected by heparin in case of the bead-capture procedure. In case RNA is isolated by commonly used spin column procedure, clear interference by heparin contamination is observed, which can be corrected by treatment with 6 IU heparinase 1 during cDNA synthesis (Supplementary Figure S12).

#### 3.4.2. Significant Differences in microRNA Levels are Found Among Serum and Plasma Samples

**Figure 9:**
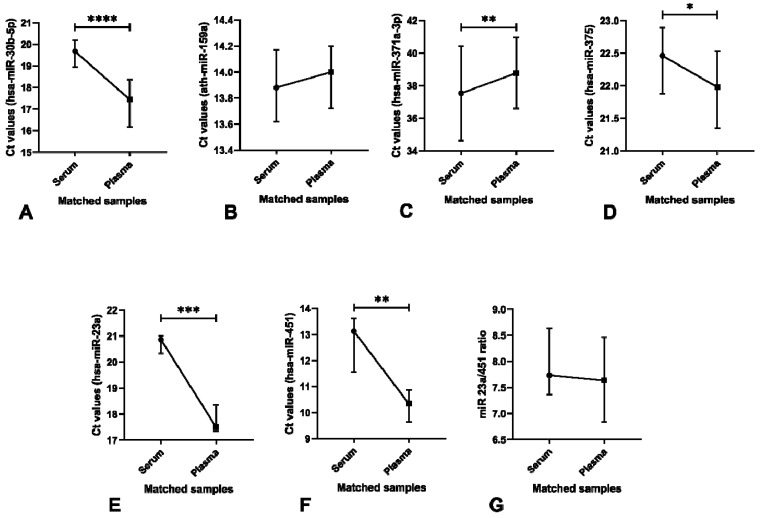
Ct values for microRNAs in matched serum and plasma samples from normal males. Ct values of normalizer hsa-miR-30b-5p (**A**), spike-in ath-miR-159a (**B**), and target microRNAs hsa-miR-371a-3p (**C**) and hsa-miR-375 (**D**) in matched serum and plasma samples from normal blood donors (*n* = 66); Ct values of hemolysis controls hsa-miR-23a-3p (**E**) and hsa-miR-451a (**F**), and respective miR-23a/451a ratio (**G**) in matched serum and plasma samples from normal blood donors (*n* = 11).

When comparing matched serum and EDTA plasma samples from normal male blood donors, a significant difference in the Ct values of the normalizer miR-30b-5p was depicted (*p* < 0.0001) (Figure 9A). Plasma samples showed significantly lower Ct values when compared to matched serum samples. No significant differences were found for the spike-in ath-miR-159a, despite some variation (Figure 9B). Additionally, Ct values of miR-371a-3p were significantly higher in plasma samples when compared to serum (*p* = 0.0048) (Figure 9C). On the contrary, plasma samples showed significantly lower Ct values of miR-375 when compared to matched serum samples (*p* = 0.0137) (Figure 9D).

No plasma samples were considered to have hemolysis by the cutoff defined by us, with only one serum sample being above the cutoff. Plasma samples showed significantly lower Ct values for miR-23a and miR-451a when compared to serum samples (*p* = 0.001 and *p* = 0.0029, respectively) (Figure 9E–F); however, no significant differences in the miR 23a/451a ratio among matched serum and plasma samples were depicted (Figure 9G). 

#### 3.4.3. MicroRNA Profiles of Control Serum and Cerebral Spinal Fluid Samples are Distinct

Although normal serum and plasma have been investigated for microRNA profiling, this has not been performed for normal CSF samples so far. Therefore, microRNA profiling was performed on four control CSF samples (TLDA card A), i.e., without neoplastic or inflammatory disease. Additionally, 16 normal male sera samples were also subjected to the same microRNA profiling (TLDA cards A + B). Of the 384 microRNAs investigated in the CSF samples, 307 (80%) were not detected in any of the samples. Only 16 microRNAs were consistently detected in all four samples (Supplementary Figure S13A). In contrast, of the 384 (plate A) and 764 (plates A + B) microRNAs investigated in the sera samples of healthy males, only 131 (34%) and 369 (48%) were absent from all samples, respectively. Thirty-eight and 50 microRNAs (10% and 7%) were detected in all 16 sera samples, respectively (Supplementary Figure S13B,C). The commonly used normalizer in serum samples miR-30b-5p was barely detected in 2/4 CSF samples (Ct values of 33.4 and 33.9), indicating its inappropriateness for normalization purposes. Raw data is provided in Supplementary File 2.

## 4. Discussion

Liquid biopsies are progressively conquering their way into the clinical setting. The quest for finding the optimal biomarkers for detection in liquid biopsy setting is on, but many challenges need to be overcome [55]. In fact, the amount of studies reporting promising biomarkers in limited tissue-based cohorts substantially exceeds the number of liquid biopsy-based validation works. Appropriate control samples, sufficiently large cohorts, pre-analytical variables and different pipelines for quantification are among the reasons for this, sometimes resulting in studies showing controversial and non-reproducible results. Therefore, there is a need for a biological model of predicting candidate biomarkers specifically to be pursued in liquid biopsy setting.

In our work, the miR-371/373 cluster and miR-367 were indeed amongst the most represented in all cell lines and respective media, underscoring that our in vitro model is able to identify the relevant microRNAs that already proved their value as biomarkers of the disease (Figure 1A,B). Our data also put in evidence that not all microRNAs are secreted with the same efficiency and that selectivity for retaining inside the cells (possibly fulfilling a biological role) does occur (Supplementary Figure S1). Indeed, when we look at the whole microRNA profiling of cell lines and respective conditioned media, we notice a significant number of non-secreted microRNAs. This is even more remarkable after excluding the numerous microRNAs already present in the fetal calf serum used for cell culturing (Figure 1C). This evidences the need for considering appropriate controls for experiments [56], which is not always done and will result in false positive findings.

The remarkable fast level of presence, in spite of low number of cells, and stability of miR-371a-3p levels in conditioned media suggests that a regulatory mechanism for this microRNA secretion exists, that renders it independent of external conditions (Figure 2A,B). We hypothesize that a protecting packaging mechanism might contribute to these findings. Indeed, our observations (Figure 2C) suggest that miR-371a-3p is present in exosomes (like demonstrated for other microRNAs [57]), given its consistent presence at similar levels in the whole and exosomal microRNA fractions; this finding should be confirmed in further studies and for other relevant targets and cell lines. Also, the amount of EVs was found to associate better with miR-371a-3p levels than the simple number of cells in culture, further strengthening this idea. Levels of miR-371a-3p were lower in exosome fractions isolated via CD63+ immunoprecipitation, in line with previous findings [58] showing that CD63 is not equally present in all exosome fractions. All in all, our data shows that the protocol will not increase in value by including an exosome purification step. This finding expands our knowledge on the mechanisms of secretion of these relevant microRNAs specifically in GCTs.

Hemolysis is a factor known to influence microRNA detection in serum/plasma samples [59]. Several methodologies for measuring hemolysis burden in serum samples have been described, with the so-called “miR-23a/451a ratio” being reported as the most accurate [42]. However, the work of Shah and collaborators differs from ours in various ways, including cohort size, microRNA extraction and quantification methods. Hence, it is fair to assume that the same cutoffs determined by the authors are not necessarily applicable in our workflow. Indeed, hemolysis did not show significant direct impact on the microRNA isolation procedure (denoted by ath-miR-159a) nor on the specific target assay miR-372a-3p, which contrasts with the findings of Myklebust and coworkers [43]. An effect on the normalizer miR-30b-5p levels was depicted; however, such effect was minor and seen mainly at the expense of cases with severe hemolysis, with visual scores of 4–5 (Figure 8), which were absent in our study cohorts. When applying the new miR-23a/451a cutoff determined by us in a second validation cohort, we found no significant differences in distribution of Ct values of spike-in, normalizer nor the specific targets miR-371a-3p/miR-375. Importantly, we believe the major reason for the little impact of hemolysis on specific targets in our study is the distinct microRNA isolation procedure: we have performed a magnetic bead capture of microRNAs, which are isolated and purified from the serum contents, eliminating the potentially harmful effects of molecules such as hemoglobin or heparin, which are known to inhibit the PCR reaction [60,61,62,63]. Indeed, we have witnessed this effect in RNA isolated from kidney preservation fluids (described in [54]) containing heparin contamination, a commonly used anticoagulant. Our new results again demonstrate that in the bead-based capture no inhibitory effect of heparin was observed and heparinase 1 digestion did not reduce the Ct values of both endogenous and spiked-in microRNAs (Supplementary Figure S12). This strengthens the advantages of this established pipeline when aiming to quantify microRNAs in liquid biopsy setting.

Evaluation of matched serum/plasma samples from the same patients depicted significant differences in the amount of the commonly used endogenous reference microRNA, miR-30b-5p, with plasma samples showing significantly lower Ct values. Significant differences in the amount of target microRNAs were also depicted (Figure 9). This knowledge is of particular importance as it may be a potential problem in mixed cohorts comprising both serum and plasma samples, again supporting the idea that mixed cohorts of both body fluids are not advisable. We hypothesize this could be due to different compositions of both fluids, both a combination of the relative proportion of fluid volume and the amount of clotting factors present in plasma samples, which might result in microRNAs sticking to them, hence escaping quantification [64]. The higher total volume of serum could explain the higher Ct values (i.e., lower levels) of miR-30b-5p when compared to plasma. The reason for observing higher Ct values for miR-371a-3p in plasma is not known, although we stress that it follows the same tendency observed for ath-miR-159a (although the latter was not statistically significant) and hence might reflect slight differences in the microRNA purification step. Overall, these results suggest that both plasma and serum can be reliably used for microRNA analyses, as previously demonstrated, but should not be compared to one another in mixed cohorts.

MicroRNA profiling of CSF samples deemed to be negative for neoplastic disease and inflammatory disease have not been reported thus far. Murray and collaborators [29] described a pipeline for quantifying microRNAs in CSF samples from pediatric GCT patients, including in their cohort four CSF samples. As controls of the experiments, five sera samples from pediatric individuals (three females and two males) were included. Our data further extends knowledge on this matter; when comparing the microRNA profiling of both normal CSF and sera samples (control subjects) we observed that a much higher proportion of tested microRNAs were absent in CSF samples when compared to sera. In CSF, 80% of microRNAs were absent in all four samples, compared to 34% in sera. Our data indicates significant differences between microRNAs present in the normal bloodstream compared to CSF, possibly due to their difference in origin [65,66]. All in all, our data stress the specificity of finding elevated levels of clinically relevant microRNAs in CSF samples for diagnostic purposes, including the use of normalization for which miR-30b-5p is not appropriate, and an alternative must be determined. 

Moreover, one must take into account the potential for microRNA decreasing (and its timing) after surgery. Our data on microRNA measurements after orchiectomy suggests that, indeed, the most reliable microRNA among the tested ones is the miR-371a-3p, which shows a steady decrease after orchiectomy and exhibits a very short half-life (<4h in our study), showing a superior profile compared to other targets (Figure 5), in line with earlier findings [25]. The fluctuations observed (in one single patient) for miR-372-3p and miR-367-3p are likely due to unspecificity of the assays used, as demonstrated by our experiment when various RT-primers were combined in pools. miR-372-3p is in a microRNA cluster together with miR-371a-3p and miR-373-3p, so cross-reaction is plausible. For miR-375 no data is available to suggest unspecificity; however, this target was found anyways to be non-informative in all the settings tested for germ cell tumors, and fluctuations may simply reflect this issue.

Similar to our in vitro findings, the in vivo mouse model further confirms the ability of identifying relevant biomarkers of the disease. Indeed, the miR-371/373 cluster and miR-367 (among others) were predicted to be most informative in plasma samples for discriminating malignant disease (Figure 3A). One of the major quests in the field of GCTs is the finding of a biomarker specific of the teratoma histology, namely one that allows the detection of residual mature/benign teratoma, for which treatment approach might differ. When focusing on microRNAs positive in these tumor xenografts, indeed most of them are not amenable to detection in plasma; however, a handful of microRNAs are pointed out by our model, namely the miR-885-5p, which could be of interest in this context (Figure 3B). Again, the relevance of using appropriate controls is underscored by the number of candidate microRNAs discarded due to their expression in normal mouse tissues/plasma (mouse-specific microRNAs instead of tumor-specific microRNAs). Based on this we pursued targeted analysis attempting to validate these markers in clinical samples. On the post-chemotherapy RPLND patient cohort, all three microRNAs were in the detection range, with no significant differences in relative levels among cases of viable tumor, teratoma or solely fibrosis/necrosis, which limits the value of these markers in this specific clinical scenario. Detection of these markers at similar levels in both teratoma and fibrosis/necrosis cases is intriguing and deserves further investigation in future studies. miR-885-5p has been shown to be a strong activator of the p53 pathway, inducing apoptosis, senescence and cell cycle arrest [67]. This makes this target very appealing from a biological point of view, since p53 pathway is activated specifically in the (miR-371a-3p-negative) teratoma [68], as opposed to the other (malignant) GCT components, which show clear upregulation of miR-371a-3p, leading to inactivation of the p53 pathway [69]. This shift in microRNA expression and impact on p53 pathway might shed light into the distinct biology and clinical behavior of these tumors. This is also in line with our findings of absence of miR-885-5p in NCCIT cells, which show absence of functional p53, while it is present in NT2 cells, where p53 is active. Also, tumor cells in necrosis (and apoptosis) after chemotherapy might be a source of miR-885-5p, explaining its expression in all RPLND samples, as some degree of necrosis/apoptosis is always present in every post-chemotherapy metastatic mass. The finding of miR-885-5p to associate with fetal growth and sperm count also seems to demonstrate an association with development, which fits the developmental model for GCTs [70,71]. Meanwhile, miR-448 has been described as inhibiting cell proliferation and invasion in several tumor models [72,73,74], but its role in this specific context is still unclear. However, despite being of limited use in this specific discrimination, these microRNAs were found to be significantly upregulated in serum samples of patients with teratoma histology when compared to normal males, allowing for a good discrimination among the two groups, which fully confirms the prediction of the in vivo model. Nevertheless, our attempt to validate these biomarkers was pursued in the specific context of post-chemotherapy RPLND masses and in a limited number of subjects, deserving for sure further validation in larger studies, to accurately assess the discrimination performance of such microRNAs, including the ability to follow-up patients with primary teratoma of the testis, both adult and pediatric. Inability to detect the teratoma histological subtype has been one of the few critics appointed to miR-371a-3p as a disease biomarker. Specifically, miR-375 was suggested to solve this gap, however shown in this study not to fulfill the necessary requirements for this purpose (which is in line with recently published data [75]), although miR-885-5p and miR-448 might be informative. In addition, this is of relevance as well in the context of regenerative medicine, in which possible formation of (benign and malignant) tumors is a significant limitation in clinical application [50]. 

## 5. Conclusions

**Figure 10:**
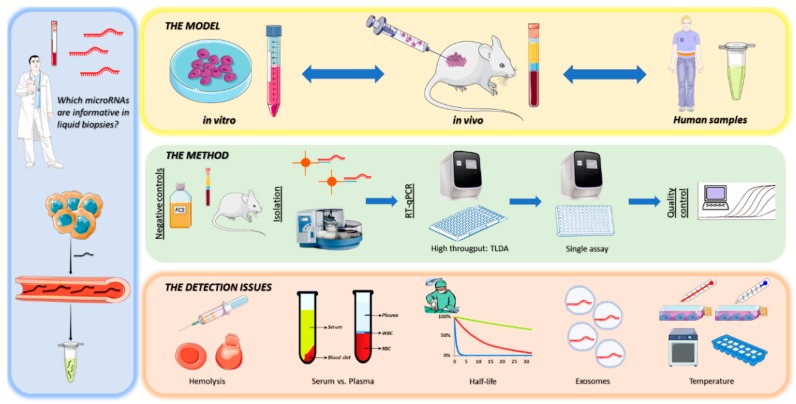
Systematic overview of the (combined in vitro and in vivo) identification model for microRNAs in liquid biopsies. (left panel) MicroRNAs are secreted by tumor cells and released into the bloodstream, therefore they could possibly be detected in liquid biopsies; (upper panel) In order to predict which microRNAs are informative in liquid biopsies, a combined in vitro and in vivo identification model was set up. In vitro: cell lines + conditioned media. In vivo: xenograft mice + plasma samples. Human samples: patient cohort studies in serum/plasma/CSF; (middle panel) Methods used for identifying microRNAs. Appropriate negative controls should be used depending on the context: fetal calf serum, normal plasma/serum/CSF samples and normal mice. Bead-captured-based microRNA-isolation assures good results and is less troubled by detection issues such as hemolysis. High throughput strategies followed by targeted-assay validation are warranted. Analysis and quality control steps are crucial to assure reproducible results; (bottom panel) In parallel, there are several detection issues to take into account when identifying microRNAs. MicroRNA levels could be influenced by hemolysis, as they are released from ruptured erythrocytes. Differences between Ct values of specific target assays exist between serum and plasma, so mixed cohorts are troublesome. MicroRNAs should have a steady decrease after surgery rather than a fluctuating expression level over time. Exosomes are a major means of microRNAs secretion. Temperature could also possibly influence the dynamics of microRNA secretion, although it seems to be a rather stable process.

To conclude, our combined in vitro and in vivo identification model (Figure 10) was able to predict the most relevant microRNAs in GCTs, in line with previous observations, and took into account various pre-analytical variables, expanding our knowledge on these tumors. The miR-371a-3p stands as the most informative biomarker for these tumors, and miR-375 does not fulfil the clinical need for detection of mature teratoma, making combination of both markers non-advantageous. Importantly, for efficient implementation in the clinic, there is a need to elaborate a standardized pipeline for its analysis, making use of the appropriate controls, uniformizing the normalization procedure and adapting to the sample type in question. In summary, we demonstrate that the model is informative to identify relevant microRNAs in a liquid biopsy setting, and could be extended to other tumor/disease models.

## 6. Patents

A patent application has been filed covering the finding of using miR-885-5p and miR-448 as molecular markers for teratoma (and contradicting effect of miR-885-5p on the P53 pathway compared to miR-371a-3p).

## Figures and Tables

**Table 1:** Ct-values for miR-371a-3p, miR-372-3p, miR-373-3p and miR-367 in cell lines and matched conditioned media.

**TCam-2**		
microRNA	Ct values	
	Cells	Media
hsa-miR-371a-3p	25.4	25.9
hsa-miR-372-3p	19.8	20.9
hsa-miR-373-3p	27.3	24.7
hsa-miR-367	24.6	26.0
**NT2**		
microRNA	Ct values	
	Cells	Media
hsa-miR-371a-3p	33.4	33.2
hsa-miR-372-3p	28.4	40
hsa-miR-373-3p	40	N/A*
hsa-miR-367	17.4	23.7
**NCCIT**		
microRNA	Ct values	
	Cells	Media
hsa-miR-371a-3p	31.3	33.0
hsa-miR-372-3p	25.9	26.6
hsa-miR-373-3p	29.8	40
hsa-miR-367	20.9	27.2
**2102Ep**		
microRNA	Ct values	
	Cells	Media
hsa-miR-371a-3p	27.1	26.7
hsa-miR-372-3p	21.5	22.4
hsa-miR-373-3p	30.4	26.1
hsa-miR-367	26.5	24.6

* Not applicable—only cases detectable in cells were considered.

**Table 2:** Relation between cell count, extracellular vesicles amount and miR-371a-3p.

Cells	Cell count per mL (confluent) × 104	EVs per mL (EVQuant)	miR-371a-3p Ct values
**TCam-2**	391	2.99E + 10	29
**NCCIT**	932	2.04E + 10	32

Abbreviations: EVs—extracellular vesicles.

**Table 3:** Performance parameters for miR-885-5p and miR-448 in discriminating teratoma from normal male individuals.

Context	Sensitivity (%)	Specificity (%)	PPV (%)	NPV (%)	Accuracy (%)
miR-885-5p	86.7	83.3	92.9	71.4	85.7
miR-448	80.0	83.3	100	62.5	81.0
Both miR-885-5p and miR-448 above defined cutoff	73.3	83.3	91.7	55.6	76.2
Either miR-885-5p or miR-448 above defined cutoff	93.3	83.3	93.3	83.3	90.5

Abbreviations: PPV—positive predictive value; NPV—negative predictive value.

## Data Availability

All data generated or analyzed during this study are included in this article and its supplementary information files.

## Supplementary Materials

The following are available online at https://www.mdpi.com/2073-4409/8/12/1637/s1: Figure S1. Proposed model for the microRNA secretion process. When microRNAs are detected in cell lines as well as in conditioned media, they are likely to be secreted and can possibly be detected in liquid biopsies. However, when microRNAs are detected in cell lines only, microRNAs are not proposed to be secreted and could show a biological function only. When microRNAs are secreted by tumor cells and released into the bloodstream, detection in liquid biopsies and identification of relevant microRNAs in human patient samples could be possible. Figure S2. Experimental outline of microRNA isolation, comparing total microRNA isolation and exosomal microRNA fractions. Figure S3. Mouse xenograft model. Cell lines (TCam-2, NT2, NCCIT and 2102Ep), human pluripotent stem cells (hPSCs) and induced pluripotent stem cells (IPS) were injected subcutaneously into immunodeficient mice. Tumor xenografts grew until a maximum size of 2cm^3^, after which tissue was collected (endpoint tissue sample). Additionally, plasma samples were taken (endpoint plasma sample). Figure S4. Rationale and workflow performed for testicular germ cell tumor patients undergoing chemotherapy and retroperitoneal lymph-node dissection (RPLND) after orchiectomy. Serum samples from TGCT patients were collected in three different time points: pre-chemotherapy; post-chemotherapy and pre-RPLND; and post-RPLND. The miR-371a-3p and miR-375 were determined. Prediction of histology at RPLND is relevant since it can impact on treatment strategy. Figure S5. Potential impact of methodology applied on microRNA isolation. (**A**) Waterfall plots showing ∆Ct values of miRNAs (Ct values in bead-based isolation—Ct values with non-bead-based extraction from total RNA) in cell lines (TCam-2, NT2, NCCIT and 2102Ep). (**B**) ∆Ct values (Ct values for samples without pre-amplification—Ct values of same samples with pre-amplification step, upper panel) with a median of 10.25 (bottom panel) in the 2102Ep cell line. Notice that in both A and B ∆Ct values are very stable. Figure S6. Distribution of microRNAs in cell lines, matched media and ∆Ct values. (**A–C**) TCam-2; (**D–F**) NT2; (**H–I**) NCCIT; (**J–L**) 2102Ep. Figure S7. Amplification of miR-372-3p after RT-qPCR performed for TCam-2 cell line with several RT-primer pools. The x-axis represents the several pools and the RT-primers that were omitted in each condition. Results are depicted as 40-Ct format. Blue—template; red—no template control. Figure S8. Detection of miR-375 and miR-371a-3p in cell lines/media and plasma samples from mouse tumor xenografts/controls. Bar graphs showing Ct values of miR-375 (**A**) and miR-371a-3p (**C**) in cell lines (TCam-2, NCCIT, NT2 and 2102Ep) and matched media; box plots showing Ct values of miR-375 (**B**) and miR-371a-3p (**D**) in plasma samples from mouse xenografts with different histology (benign teratoma, malignant) and control normal mice. Figure S9. miR-375 as serum biomarker for testicular germ cell tumors in the context of post-chemotherapy retroperitoneal lymph node dissection (RPLND). Boxplots showing relative miR-375 expression in (**A**) different histological TGCT subtypes (fibrosis/necrosis, teratoma, viable tumor) at RPLND; (**B**) different time points: pre-chemotherapy, post-chemotherapy and post-RPLND; (**C**) and according to disease stage (patients vs. normal male controls). Because controls were included in the comparison, the reference sample was the one showing the highest Ct value in these analyses. Figure S10. Receiver operating characteristic (ROC) curve of ∆Ct_(miR23a-miR451a)_ as a marker for hemolysis and determination of the optimal threshold. (**A**) The threshold value is displayed in red. (**B**) The red line represents the maximum of the Youden’s index, which is equal to the threshold of ∆Ct = 9.15. Figure S11. Hemolysis scoring based on visual inspection and miR23a/451a ratio. Visual inspection (**A**) and hemolysis impact on Ct values of the spike-in ath-miR-159a, the normalizer hsa-miR-30b-5p and target assays hsa-miR-371a-3p and hsa-miR-375, determined by the cutoff of miR-23a/451a ratio 9.15 (**B**), of serum samples from patients undergoing chemotherapy followed by retroperitoneal lymph-node dissection (RPLND). Figure S12. Bead isolation of microRNAs is insensitive to heparin. Effect of heparin contamination in quantification of spiked-in (**A**,**B**) and endogenous (**C**,**D**) microRNAs in bead- versus column–isolated samples from preservation fluids of kidney grafts. A) cel-miR-39-3p spiked-in during lysis procedure, B) cel-miR-54-3p spiked-in during cDNA synthesis, and the two endogenous microRNAs C) hsa-miR-21-5p and D) hsa-miR-505-3p. cDNA synthesis of microRNAs from both bead- and column-isolated samples was performed in the presence (+) or absence of (-) 6 IU heparinase 1. The pre-amplification step was not applied in the case of cel-miR-54-3p. Shown is the result of four representative samples. Figure S13. MicroRNA profiling in normal cerebral spinal fluid. (**A**) compared to normal male sera samples (TLDA plate A only—(**B**)—and TLDA plates A+B—(**C**)). Numbers outside the venn diagram (not included in any ellipse) refer to microRNAs not detected in any sample. MicroRNAs of the 371/373 cluster, miR-367, miR-375 and miR-448 were not detected in any CSF samples, while miR-885-5p and miR-197-3p were detected in 2/4 samples; similarly, miR-448 was not detected in any of the 16 normal male sera, but was in the detection range in 15 and 16 of these, for miR-885-5p and miR-197-3p, respectively. Abbreviations: CSF—cerebral spinal fluid. Supplementary File 1. MicroRNAs secretion by cell lines. Heatmaps showing microRNA secretion by TCam-2, NT2, NCCIT and 2102Ep cell lines. Supplementary File 2. MicroRNA profiling of normal cerebral spinal fluid and sera samples. Table S1. Clinicopathological features of RPLND series. Abbreviations: AFP—alpha fetoprotein; β-HCG—human chorionic gonadotropin subunit beta; IQR—interquartile range; LDH—lactate dehydrogenase; LN—lymph node; RPLND - retroperitoneal lymph-node dissection. Table S2. Clinicopathological features of clinical stage I series. Abbreviations: IQR—interquartile range. Table S3—Diagnoses of the cohort of type I and type II tumors presenting in young age.
