# Supplementary material for: Identification and Validation Model for Informative Liquid Biopsy-Based microRNA Biomarkers: Insights from Germ Cell Tumor In Vitro, In Vivo and Patient-Derived Data
Source: Cells. 2019 Dec 14;8(12):1637. doi: 10.3390/cells8121637 (PMC6952794; doi:10.3390/cells8121637)
Supplement: Supplementary file 1 [file cells-08-01637-s001.zip › Supplementary Figure 13.pdf]

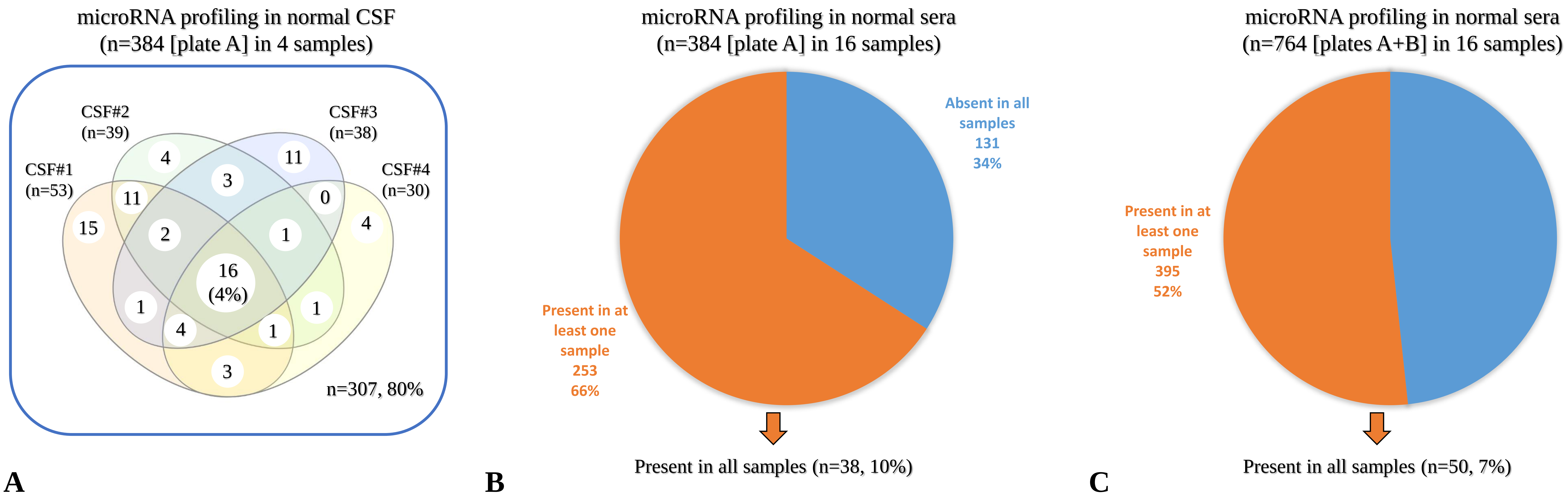
microRNA profiling in normal CSF
(n=384 [plate A] in 4 samples)
CSF#1
(n=53)
CSF#2
(n=39)
CSF#3
(n=38)
CSF#4
(n=30)
4
11
3
11
0
15
2
1
4
16
(4%)
1
1
4
1
3
n=307, 80%
A
microRNA profiling in normal sera
(n=384 [plate A] in 16 samples)
Absent in all samples 131 34%
Present in at least one sample 253 66%
Present in all samples (n=38, 10%)
B
microRNA profiling in normal sera
(n=764 [plates A+B] in 16 samples)
Present in at least one sample 395 52%
Absent in all samples 369 48%
Present in all samples (n=50, 7%)
C
