# Supplementary material for: Identification and Validation Model for Informative Liquid Biopsy-Based microRNA Biomarkers: Insights from Germ Cell Tumor In Vitro, In Vivo and Patient-Derived Data
Source: Cells. 2019 Dec 14;8(12):1637. doi: 10.3390/cells8121637 (PMC6952794; doi:10.3390/cells8121637)
Supplement: Supplementary file 1 [file cells-08-01637-s001.zip › Supplementary Table 1.docx]

**Supplementary Table 1 – Clinicopathological features of RPLND series**

| Variables | Patient cohort (n=40) |
| --- | --- |
| Age [years (median, IQR)] | 27 (23-33) |
| Laterality of primary tumor (n, %) |  |
| Right | 17/38 (44.7) |
| Left | 21/38 (55.3) |
| Multifocality in primary tumor (n, %) |  |
| Absent | 31/36 (86.1) |
| Present | 5/36 (13.9) |
| Predominant histology in primary tumor (n, %) |  |
| Embryonal carcinoma | 23/38 (60.5) |
| Seminoma | 6/38 (15.8) |
| Postpubertal-type teratoma | 4/38 (10.5) |
| Postpubertal-type yolk sac tumor | 2/38 (5.3) |
| Choriocarcinoma | 1/38 (2.6) |
| “Burned-out” tumor | 2/38 (5.3) |
| Primary tumor size [cm (median, IQR)] | 4 (2.3-5.5) |
| pT stage (n, %) |  |
| pT1 | 15/36 (41.7) |
| pT2 | 21/36 (58.3) |
| N stage (n, %) |  |
| N0 | 4/36 (11.1) |
| N1 | 4/36 (11.1) |
| N2 | 18/36 (50.0) |
| N3 | 10/36 (27.8) |
| M stage |  |
| M0 | 23/38 (60.5) |
| M1a | 14/38 (36.9) |
| M1b | 1/38 (2.6) |
| S stage |  |
| S0 | 6/38 (15.8) |
| S1 | 20/38 (52.6) |
| S2 | 7/38 (18.4) |
| S3 | 5/38 (13.2) |
| Stage groups |  |
| I | 4/39 (10.3) |
| II | 19/39 (48.7) |
| III | 16/39 (41.0) |
| Pre-chemotherapy AFP [ng/mL (median, IQR)] | 30 (5-256) |
| Pre-chemotherapy β-HCG [mUI/mL (median, IQR)] | 29 (2-1233) |
| Pre-chemotherapy LDH [U/L (median, IQR)] | 262 (187-436) |
| LN size, pre-chemotherapy [cm (median, IQR)] | 3.65 (2.0-7.6) |
| LN size at RPLND [cm (median, IQR)] | 8 (4.0-11.0) |
| LN location (n, %) |  |
| Retroperitoneal | 22/38 (57.9) |
| Retroperitoneal and chest | 16/38 (42.1) |
| RPLND histology (n, %) |  |
| Fibrosis/Necrosis | 18/39 (46.2) |
| Teratoma | 15/39 (38.4) |
| Viable tumor (non-teratoma) | 6/39 (15.4) |
| Serum samples available (n) |  |
| Pre-chemotherapy | 37 |
| Post-chemotherapy | 39 |
| Post-RPLND | 40 |

**Abbreviations:** AFP – alpha fetoprotein; β-HCG – human chorionic gonadotropin subunit beta; IQR – interquartile range; LDH – lactate dehydrogenase; LN – lymph node; RPLND - retroperitoneal lymph-node dissection.
