# Supplementary material for: Identification and Validation Model for Informative Liquid Biopsy-Based microRNA Biomarkers: Insights from Germ Cell Tumor In Vitro, In Vivo and Patient-Derived Data
Source: Cells. 2019 Dec 14;8(12):1637. doi: 10.3390/cells8121637 (PMC6952794; doi:10.3390/cells8121637)
Supplement: Supplementary file 1 [file cells-08-01637-s001.zip › Supplementary Table 2.docx]

**Supplementary Table 2 – Clinicopathological features of clinical stage I series**

| Variables | Patient cohort (n=12) |
| --- | --- |
| Age [years (median, IQR)] | 36 (30-46) |
| Histology in primary tumor (n, %) |  |
| Seminoma | 9/12 (75.0) |
| Non-seminoma | 3/12 (25.0) |
| Primary tumor size [mm (median, IQR)] | 30 (16.0-55.5) |
| Serum samples available (n) |  |
| Pre-orchiectomy | 12 |
| 4h post-orchiectomy | 2 |
| 8h post-orchiectomy | 1 |
| 10h post-orchiectomy | 1 |
| 24h post-orchiectomy | 12 |
| 48h post-orchiectomy | 12 |
| 72h post-orchiectomy | 9 |
| 96h post-orchiectomy | 1 |

**Abbreviations:** IQR – interquartile range.
