# Supplementary material for: Identification and Validation Model for Informative Liquid Biopsy-Based microRNA Biomarkers: Insights from Germ Cell Tumor In Vitro, In Vivo and Patient-Derived Data
Source: Cells. 2019 Dec 14;8(12):1637. doi: 10.3390/cells8121637 (PMC6952794; doi:10.3390/cells8121637)
Supplement: Supplementary file 1 [file cells-08-01637-s001.zip › Supplementary Table 3.docx]

**Supplementary Table 3 – Diagnoses of “type I and type II” cohort**

| Diagnosis | Patients (n=26) |
| --- | --- |
| Mature teratoma of the testis | 15 |
| Mature teratoma of the ovary | 1 |
| Mature teratoma, extragonadal (mediastinum) | 1 |
| Mixed teratoma and yolk sac tumor of the testis | 1 |
| Immature teratoma of the ovary | 1 |
| Immature teratoma, extragonadal (mediastinum) | 1 |
| Mixed teratoma and yolk sac tumor, extragonadal (pinealis) | 1 |
| Yolk sac tumor of the testis | 3 |
| Yolk sac tumor of the ovary | 1 |
| Dysgerminoma of the ovary | 1 |
| Diagnosis | **Controls (n=10)** |
| “No malignancy” | 3 |
| Hydrocephalus | 1 |
| Pericarditis | 1 |
| Scrotal haematocele | 1 |
| Lymphadenopathy | 1 |
| Vaginal atresia | 1 |
| Meconium peritonitis | 1 |
| Bronchogenic cyst | 1 |
