# Supplementary material for: Identification and Validation Model for Informative Liquid Biopsy-Based microRNA Biomarkers: Insights from Germ Cell Tumor In Vitro, In Vivo and Patient-Derived Data
Source: Cells. 2019 Dec 14;8(12):1637. doi: 10.3390/cells8121637 (PMC6952794; doi:10.3390/cells8121637)
Supplement: Supplementary file 1 [file cells-08-01637-s001.zip › Supplementary Figure 1.pdf]

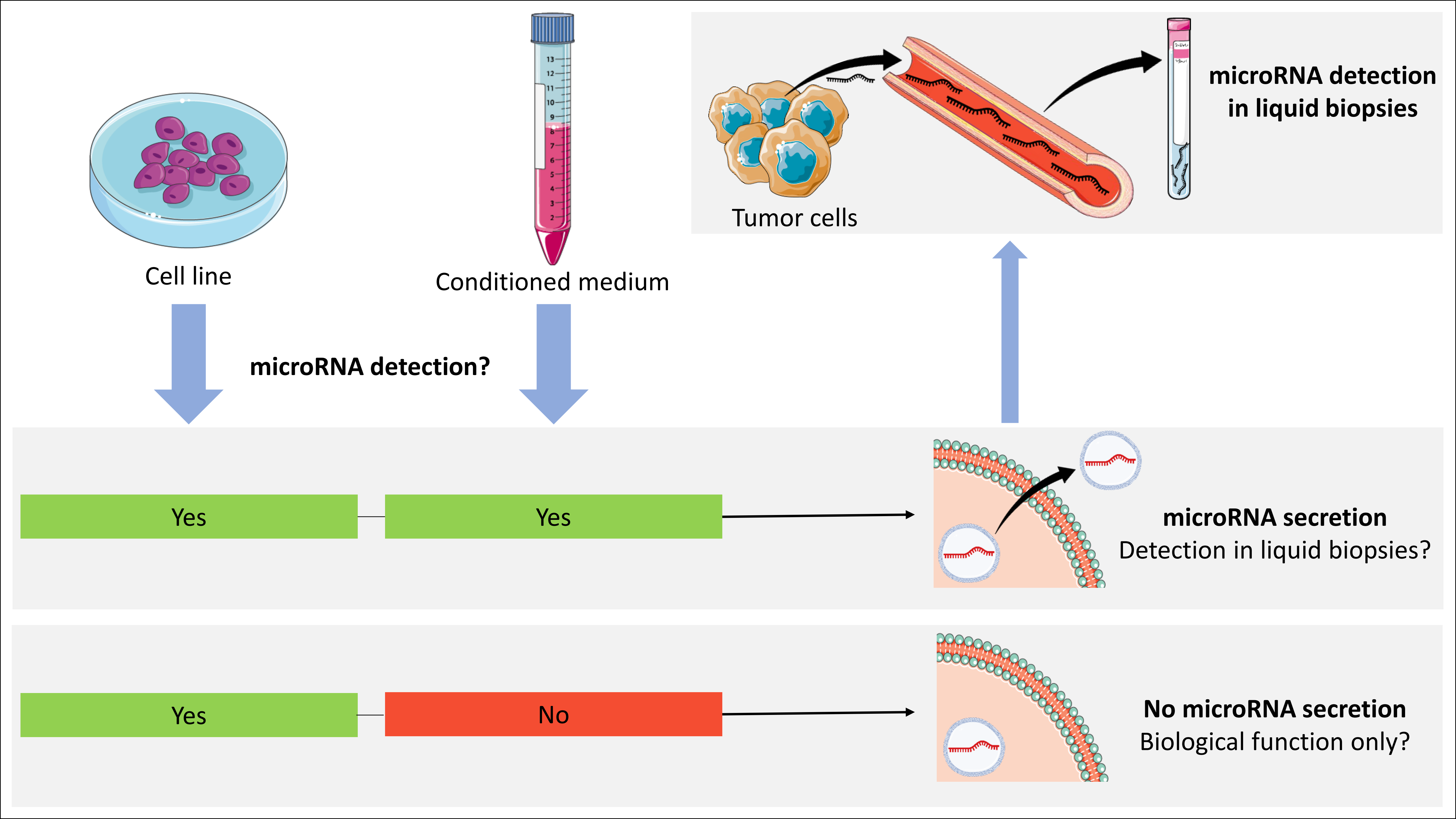

Cell line
Conditioned medium
microRNA detection?
Tumor cells
microRNA detection in liquid biopsies
Yes
Yes
microRNA secretion
Detection in liquid biopsies?
Yes
No
No microRNA secretion
Biological function only?
