# Supplementary figures and images for: Identification and Validation Model for Informative Liquid Biopsy-Based microRNA Biomarkers: Insights from Germ Cell Tumor In Vitro, In Vivo and Patient-Derived Data
Source: Cells. 2019 Dec 14;8(12):1637. doi: 10.3390/cells8121637 (PMC6952794; doi:10.3390/cells8121637)

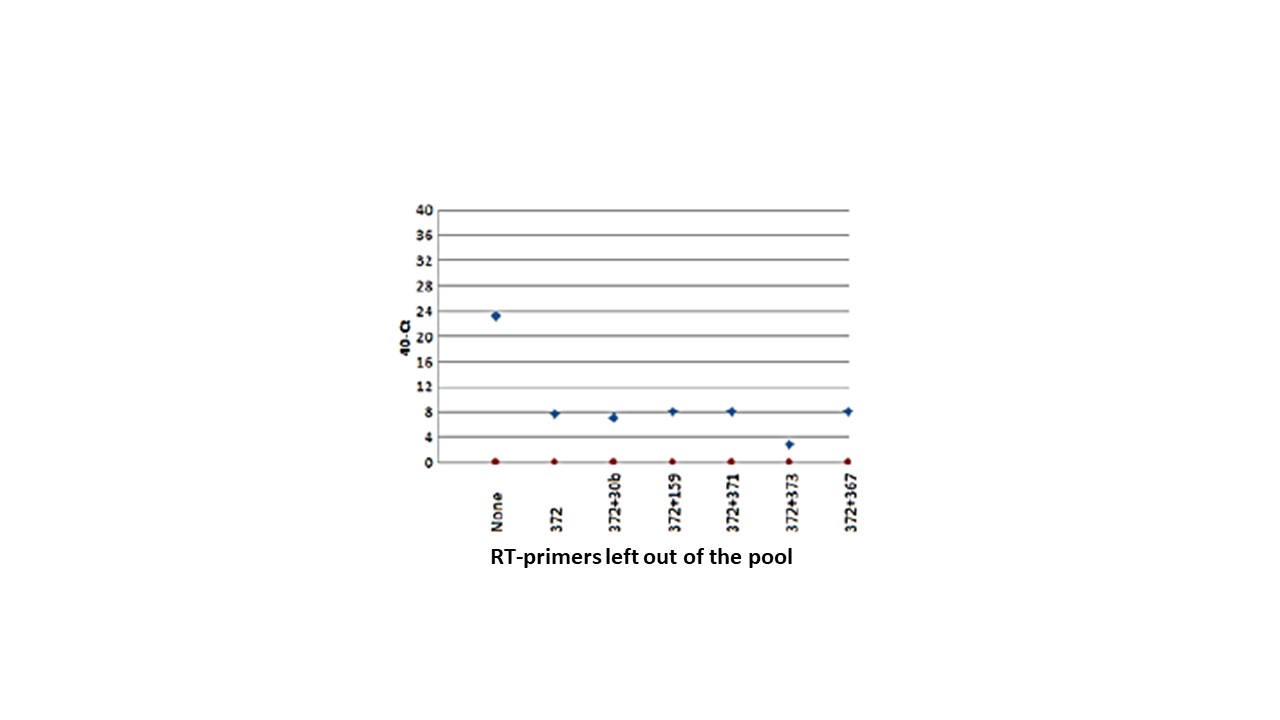

Supplement: Supplementary file 1 [file cells-08-01637-s001.zip › Supplementary Figure 7.jpg]

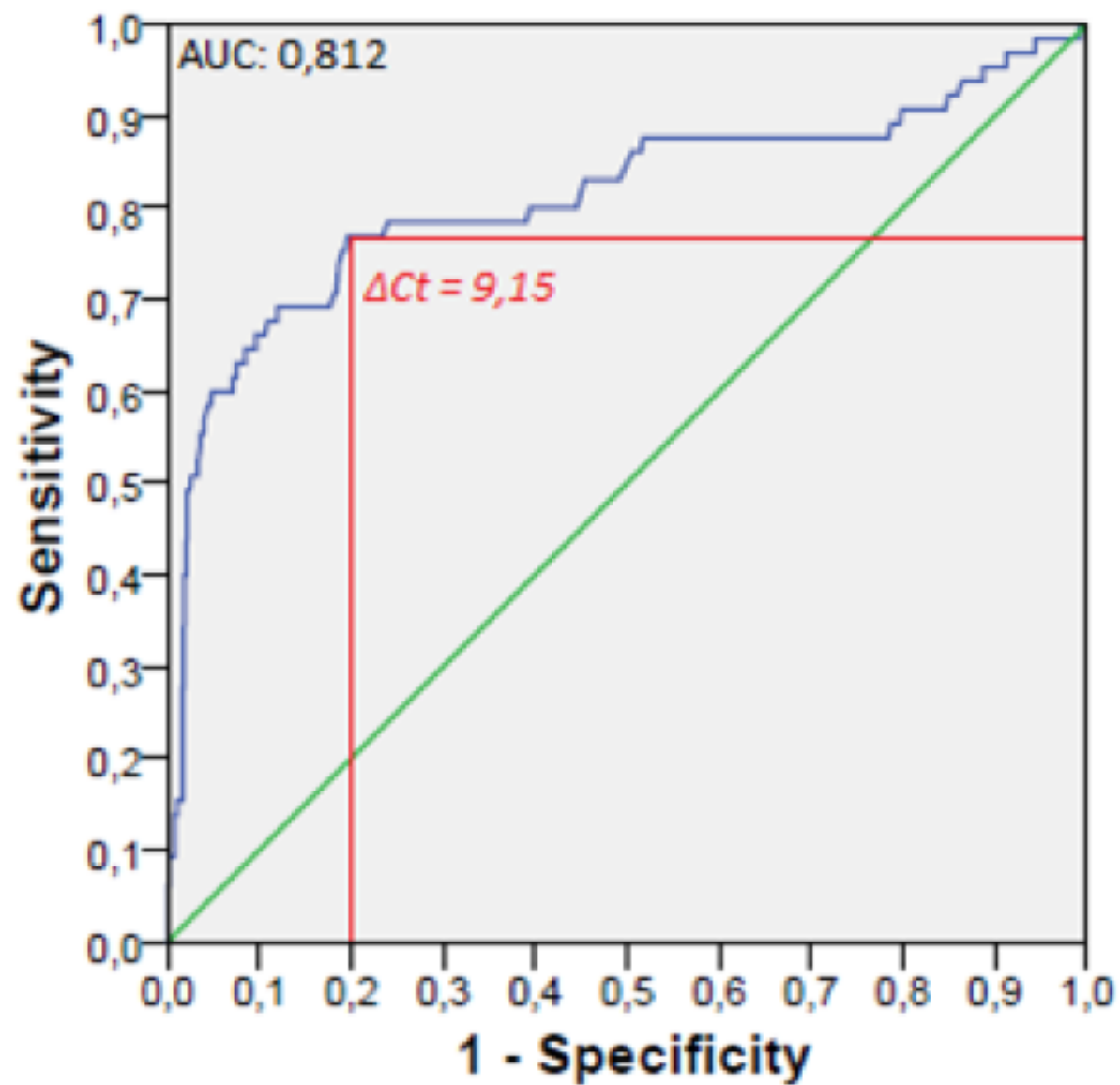

A

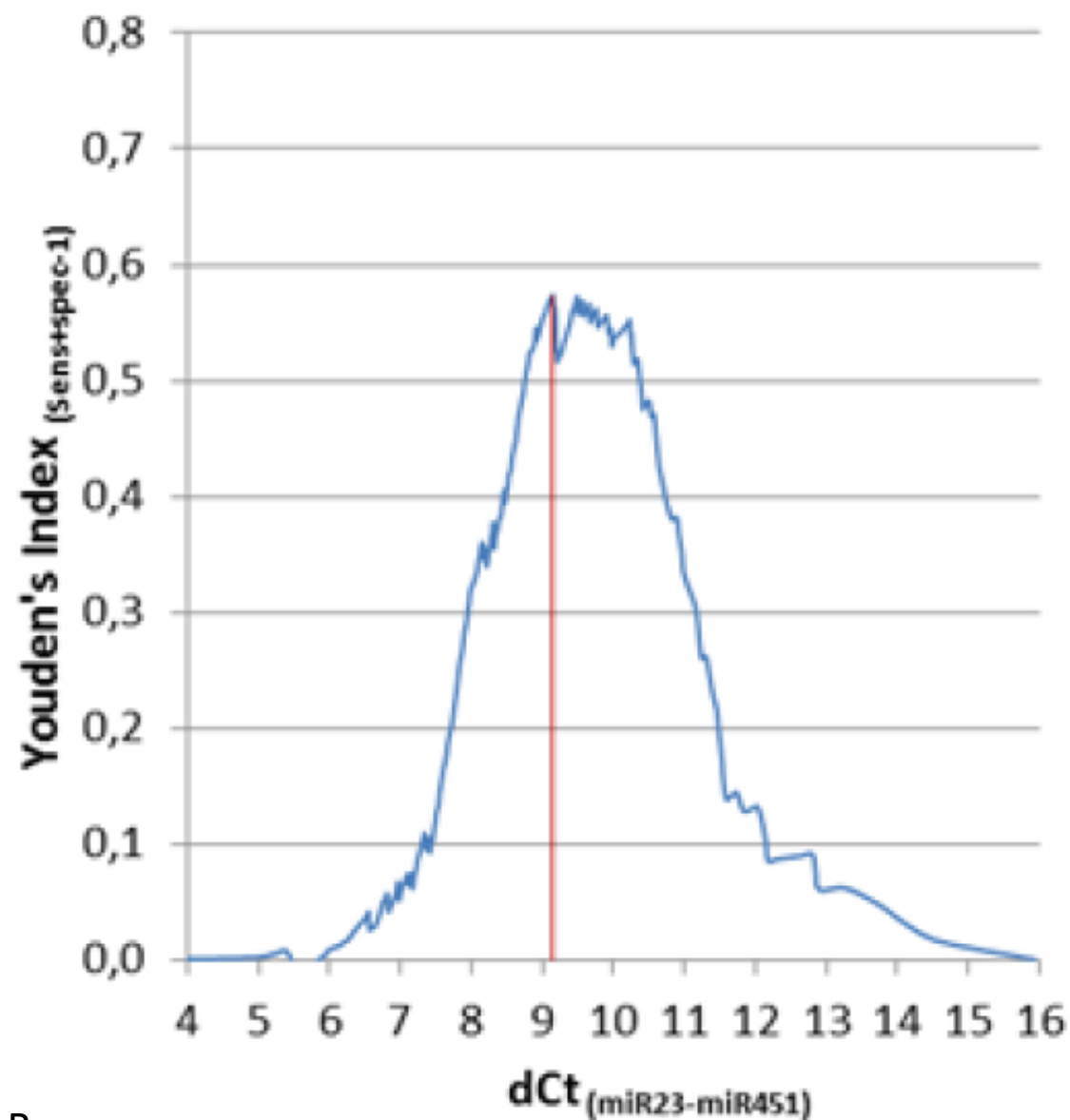

B

Supplement: Supplementary file 1 [file cells-08-01637-s001.zip › Supplementary Figure 10.pdf]

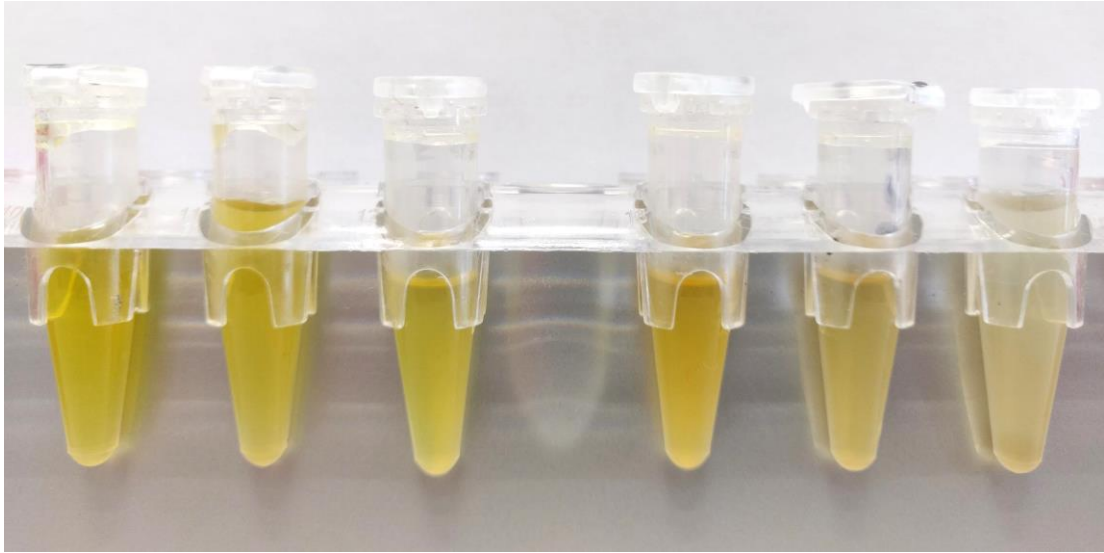

A

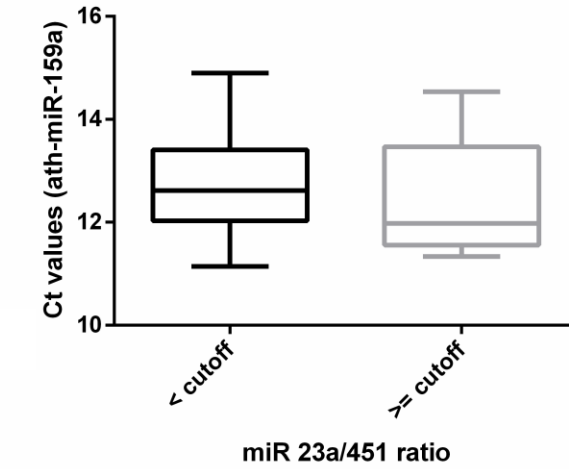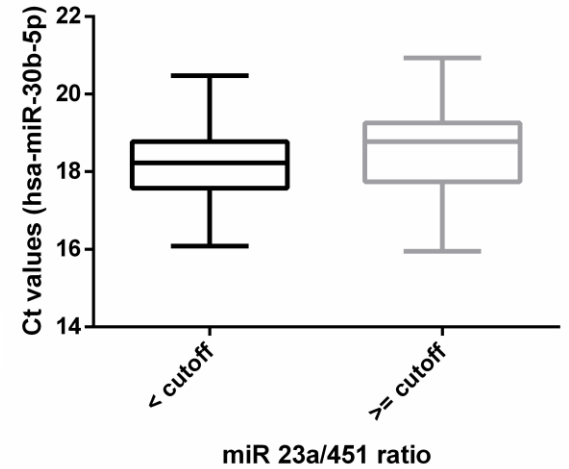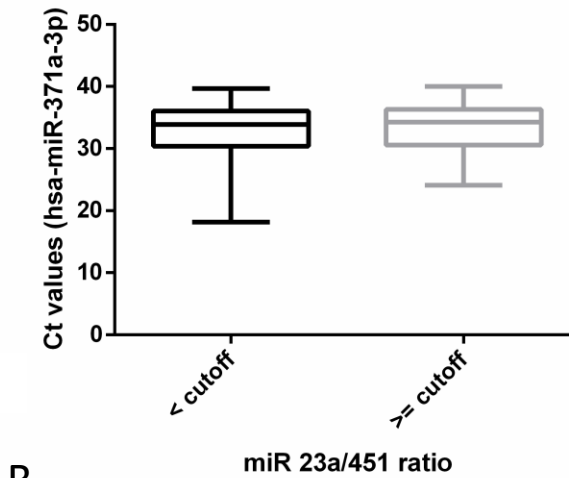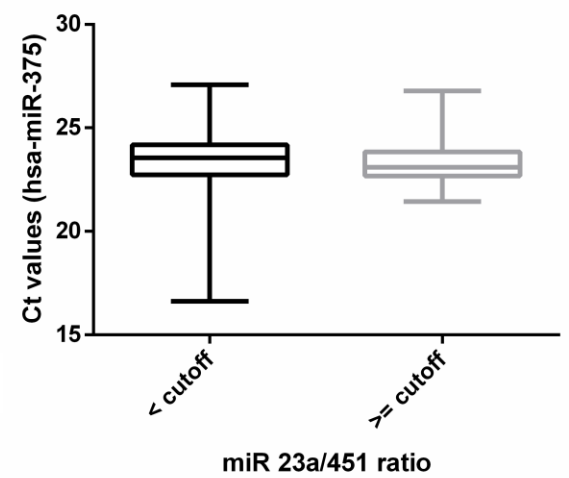

B

Supplement: Supplementary file 1 [file cells-08-01637-s001.zip › Supplementary Figure 11.pdf]

**A** Cel-miR-39-3p

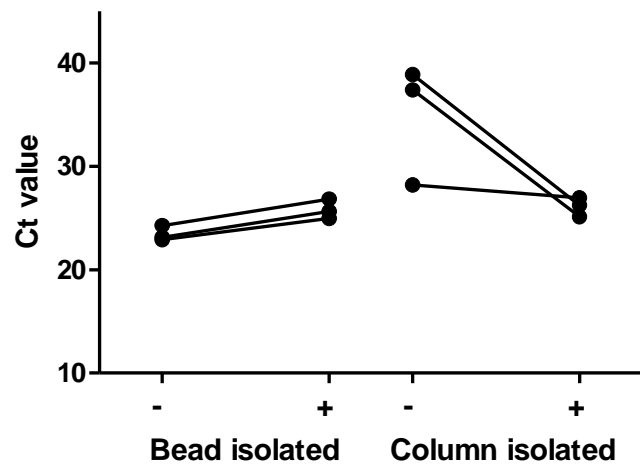

**B** Cel-miR-54-3p

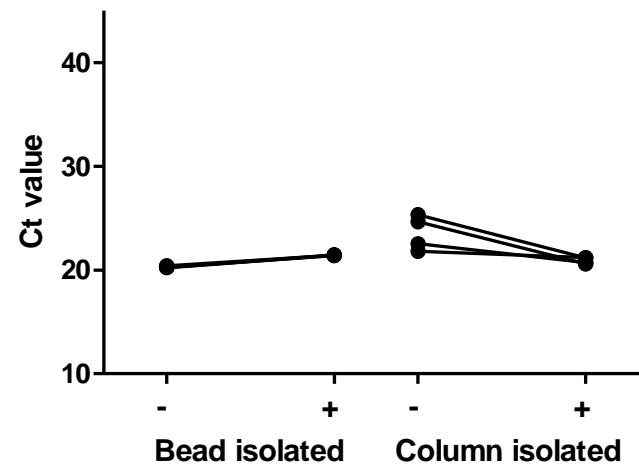

**C** Hsa-miR-21-5p

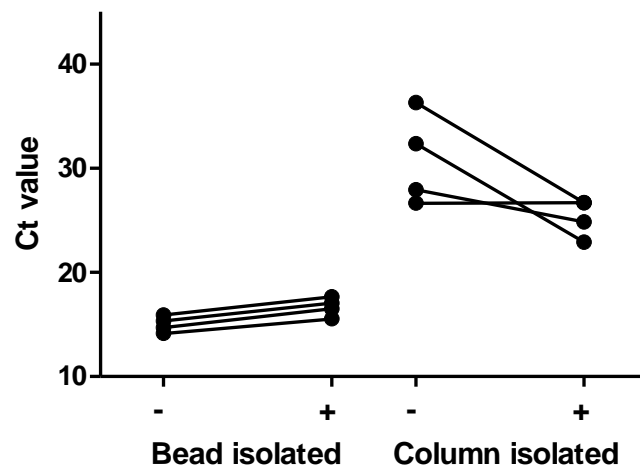

**D** Hsa-miR-505-3p

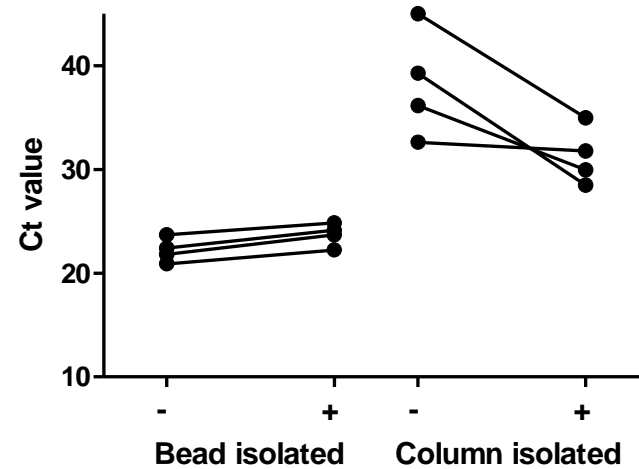

Supplement: Supplementary file 1 [file cells-08-01637-s001.zip › Supplementary Figure 12.pdf]

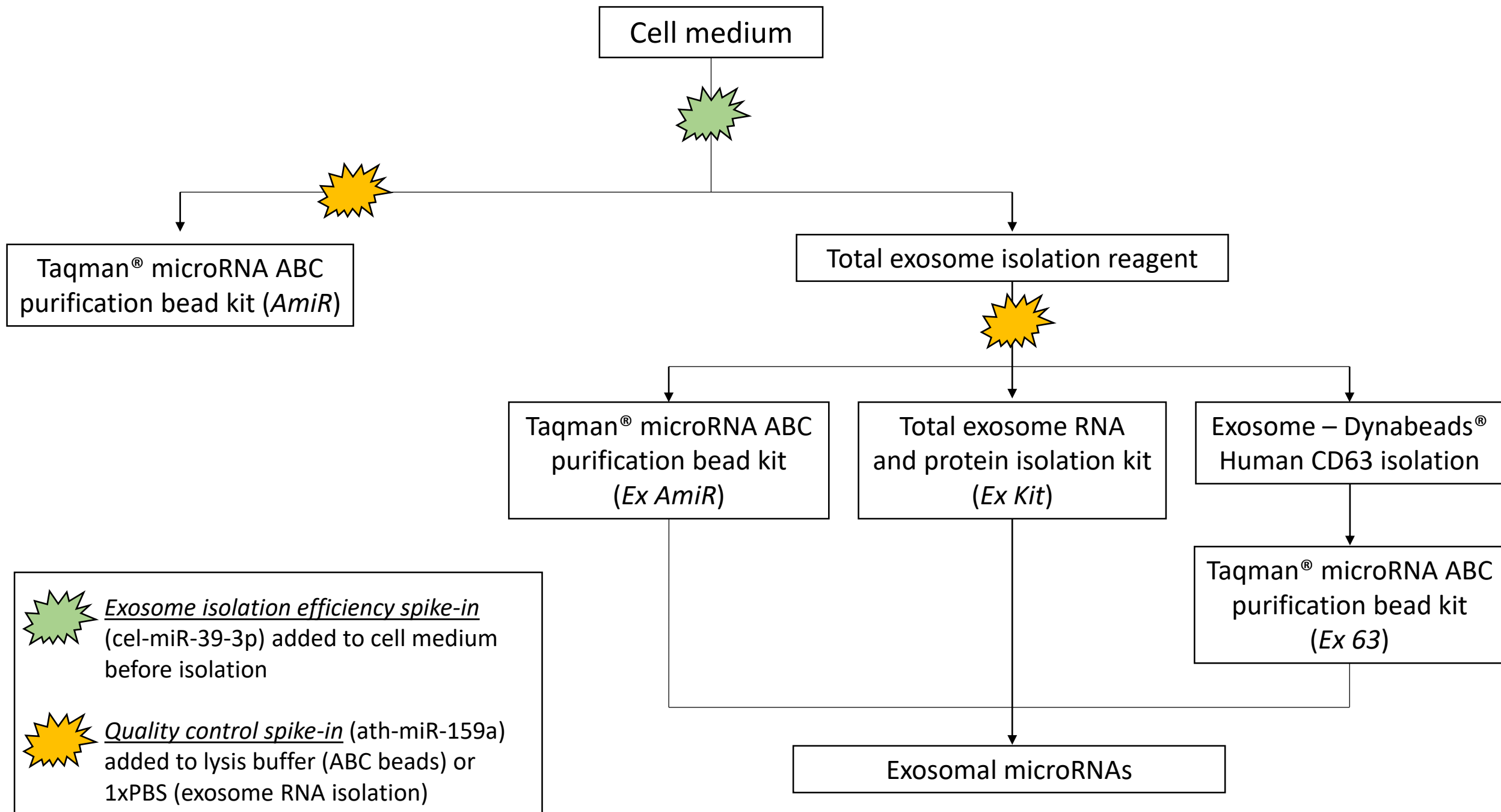

Supplement: Supplementary file 1 [file cells-08-01637-s001.zip › Supplementary Figure 2.pdf]

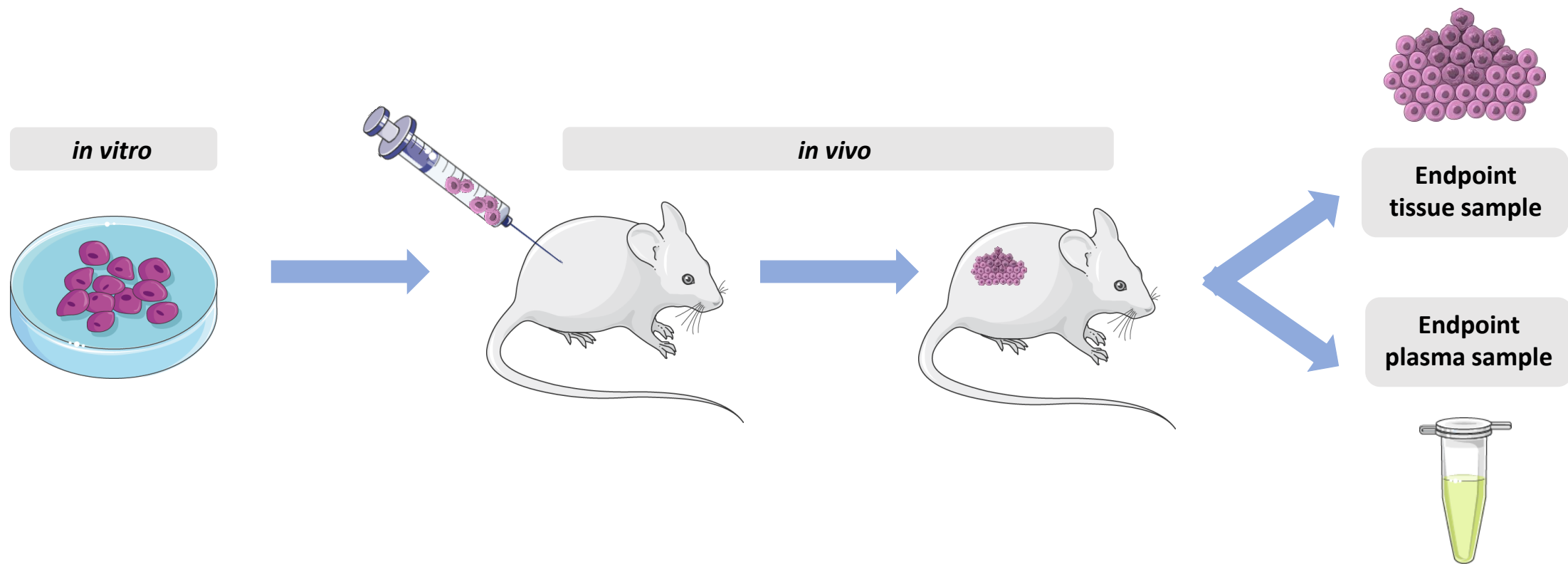

Supplement: Supplementary file 1 [file cells-08-01637-s001.zip › Supplementary Figure 3.pdf]

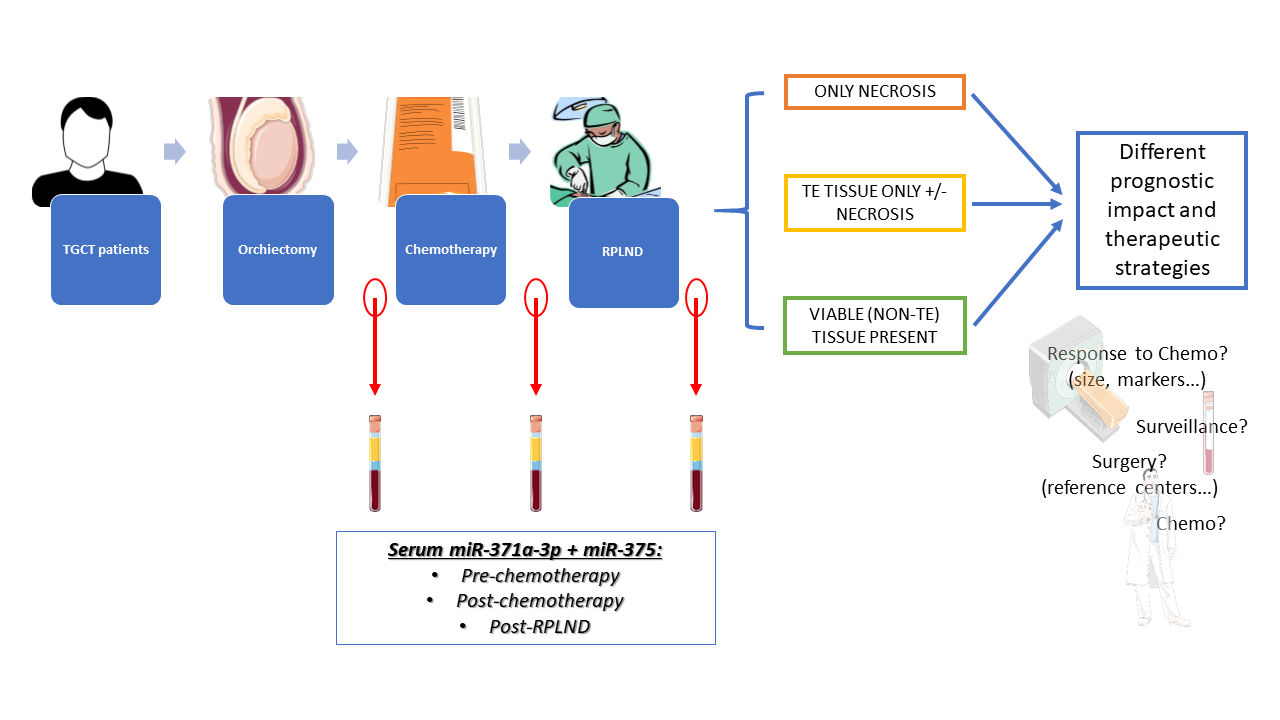

Supplement: Supplementary file 1 [file cells-08-01637-s001.zip › Supplementary Figure 4.tif]
